# Creation of Elite Rice with High-Yield, Superior-Quality and High Resistance to Brown Planthopper Based on Molecular Design

**DOI:** 10.1186/s12284-022-00563-7

**Published:** 2022-03-15

**Authors:** Manman Liu, Fengfeng Fan, Shihao He, Yu Guo, Gaili Chen, Nannan Li, Nengwu Li, Huanran Yuan, Fengfeng Si, Fang Yang, Shaoqing Li

**Affiliations:** State Key Laboratory of Hybrid Rice, Hongshan Laboratory of Hubei Province, Key Laboratory for Research and Utilization of Heterosis in Indica Rice of Ministry of Agriculture, Engineering Research Center for Plant Biotechnology and Germplasm Utilization of Ministry of Education, College of Life Science, Wuhan University, Wuhan, 430072 China

**Keywords:** Molecular design breeding, Rice, High yield, Quality, Brown planthopper

## Abstract

Breeding rice (*Oryza sativa* L.) with high yield, superior quality, desired grain shape and high resistance is the goal of breeding to meet the needs of current consumers. It is usually hard to combine multiple complex traits based on traditional breeding methods because they are frequently antagonistic to each other. However, molecular design breeding, as a novel breeding method, is an optional alternative to this challenge. To demonstrate molecular design breeding, 15 favorable genes from five parent lines were pyramided together to develop elite rice with high-yield, superior-quality, desired grain shape and high resistance to brown planthopper (BPH). The parental lines were 9311, the recurrent parent, carrying *APO1*, *Ghd7*, *Ghd8* and *Gn1a* for high yield, *GS3* and *qSW5* for grain shape, and *Wx* and *ALK* for eating and cooking quality; 1880 with *Gn8.1* for large panicles; Luo-Yu-Xiang carrying *GW7* for grain shape and *SBE3*, *SSIV2* and *SSIII* for eating and cooking quality; Luoyang6 with *Bph6* and Luoyang9 with *Bph9* for BPH resistance*.* After careful screening for the 15 targeted genes, desired phenotype and maximum genetic background from 9311, three molecular design lines with desired phenotypes, named as MD1 (Molecular design 1), MD2 and MD3 were developed. MD3 carried all 15 targeted genes, and MD1 and MD2 had 14 of the 15 targeted genes. Only *SBE3* was not introgressed into MD1 and MD2 but this had minimal impact on the gel consistency and alkali spreading value. These newly bred lines exhibited higher yield potential, better grain quality with slender grains, low amylose content, high gel consistency and alkali spreading value, and higher BPH resistance compared to the parent 9311. In this study, we successfully created three novel rice lines with high yield, superior quality and improved BPH resistance by rational molecular design. Our results demonstrate molecular design is a powerful strategy to improve multiple complex traits and will provide a reference for the future commercial rice improvement.

**Supplementary Information:**

The online version contains supplementary material available at 10.1186/s12284-022-00563-7.

## Background

The development of ‘super-rice’ varieties with high yield, superior quality and high resistance to diseases and insects is a priority for rice (*Oryza sativa* L.) breeding programs. To this end, significant effort has been invested on marker-assisted selection (MAS) for multiple traits, but to date, the reported successes centered mainly on improving resistance to biotic or abiotic stresses, such as diseases, insects and drought, but rarely on complex traits like grain yield and rice quality (Fan et al. 2015; Shamsudin et al. 2016; Wang et al. 2017; Ramalingam et al. 2020; Shailani et al. 2021). This is because most of the complex traits are controlled by quantitative loci with relatively low heritability and affected by significant genotype-by-environment interactions, linkage blocks, and complex regulatory networks (Qian et al. 2016). Therefore, breeding rice with high yield, superior quality and high resistance is challenging for rice breeders because these characters are usually antagonistic to each other (Zeng et al. 2017; Wang et al. 2018).

“Molecular Design Breeding” is a new breeding strategy for combining complex traits based on the extensive knowledge accumulated about the functional genes that regulate important agronomic traits, like grain quality and yield (Qian et al. 2016). This method greatly improves the accuracy and effectiveness of selection and accelerates pyramiding of multiple complex traits as demostrated by Zeng et al. (2017). An important prerequisite for molecular design breeding is the accumulation of genetic resources including favorable genes and important crop plant germplasm.

To date, hundreds of quantitative trait loci (QTL) and genes for high grain yield, such as *ABERRANT PANICLE ORGANIZATION 1* (*APO1*), *GRAIN NUMBER, PLANT HEIGHT AND HEADING DATE 7* (*Ghd7*), *GRAIN NUMBER, PLANT HEIGHT AND HEADING DATE 8* (*Ghd8*) and *GRAIN NUMBER 1a* (*Gn1a*), have been identified, but few of them have been widely employed in rice production (Ashikari et al. 2005; Ookawa et al. 2010; Zeng et al. 2017). Previously, we identified a high-yield gene, *GRAIN NUMBER 8.1* (*Gn8.1*), from wild rice *O. longistaminata A.*, which increases grain number per panicle up to 50%, thus it could significantly for improve rice production (Fan et al. 2017).

Rice quality mainly includes processing quality, appearance quality and eating and cooking quality. The appearance quality was mainly determined by grain shape, chalk content and transparency, while eating and cooking quality was mainly determined by amylose content, gel consistency and alkali spreading value (Tian et al. 2009). According to Chinese national standards, superior-quality rice should have suitable grain shape, low percent chalk, low amylose content, high gel consistency and high alkali spreading value, so we focused on these indicators (Zeng et al. 2017). To date, a large number of functional genes for rice appearance quality, and eating and cooking quality have been cloned, among which, *GRAIN WIDTH 7* (*GW7*) (Wang et al. 2015), *GRAIN SIZE 3* (*GS3*) (Mao et al. 2010) and QTL for *SEED WIDTH 5* (*qSW5*) (Lu et al. 2013) were targeted for grain shape, *WAXY* (*Wx*) and *STARCH SYNTHASE-III* (*SSIII*) were targeted for amylose content, *ALKALI DEGENERATION* (*ALK*), *STARCH SYNTHASE-IV-2* (*SSIV-2*) and *STARCH BRANCHING ENZYME 3* (*SBE3*) were targeted for alkali spreading value, and *Wx* and *SBE3* were targeted for gel consistency (Tian et al. 2009; Zeng et al. 2017). If it is possible to combine these genes into the same rice line, it could significantly improve rice quality.

Brown planthopper is a major pest which endangers rice by sucking the phloem sap of rice plants through its stylet mouthparts (Du et al. 2009). More than 30 brown planthopper (BPH) resistant genes have been identified from wild and cultivated rice germplasm to date (Tan et al. 2020). Among these, *Bph6* displays broad resistance to six brown planthopper biotypes (biotypes 1, 2, 3 P, S and Y), while *Bph9* confers both antixenosis and antibiosis to BPH (Zhao et al. 2016; Guo et al. 2018), thus pyramiding these two genes could create elite varieties with broad spectrum and durable resistance to BPH. Pyramiding the aforementioned important functional genes potentially provides the opportunity for us to optimize the grain yield, rice quality and resistance to brown planthopper using the molecular design breeding strategy.

The objective of this study was to demonstrate the power of molecular design breeding for developing rice varieties with high yield, improved quality and improved BPH resistance. To achieve this objective, we selected fifteen genes including *Gn8.1* for large panicles introgressed from *O. longistaminata A.* into 1880, a chromosome segment substitution line (CSSL); *APO1*, *Ghd7*, *Ghd8* and *Gn1a* for high yield, *GS3* and *qSW5* for grain shape, and *Wx* and *ALK* for eating and cooking quality in the *indica* variety 9311; *GW7* for grain shape, and *SBE3*, *SSIV2* and *SSIII* for eating and cooking quality in Luo-Yu-Xiang (Basmati mutant); and *Bph6* and *Bph9* for BPH resistance in Luoyang6 and Luoyang9, respectively. Subsequently, utilizing a combination of crossing, backcrossing and selfing along with genotyping for the 15 targeted genes and the 9311 background, we attempted to construct elite rice lines with high grain yield, superior quality and improved BPH resistance.

## Results

### Gene Selection and Phenotyping of the Parental Lines for Molecular Design

**Fig. 1:**
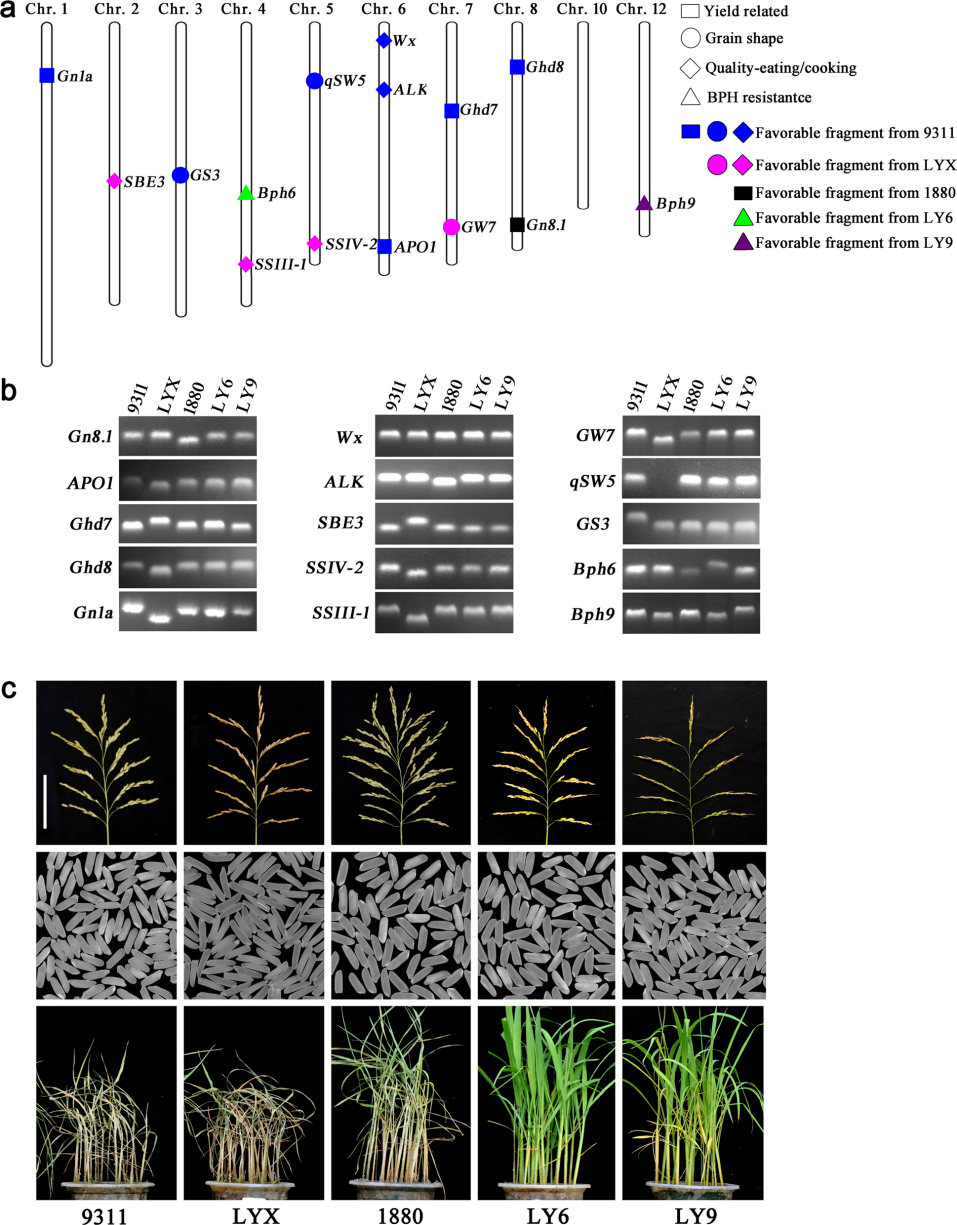
Description of target genes and parents selected for molecular design breeding. **a** The distribution of target genes on chromosomes. Square represents yield-related genes, diamond represents eating and cooking quality related genes, circle represents appearance quality related genes, triangle represents rice BPH resistance related genes; Blue indicates that the favorable fragment is from 9311, pink indicates that the favorable fragment is from Luo-Yu-Xiang (LYX), black indicates that the favorable fragment is from 1880, green indicates that the favorable fragment is from Luoyang-6 (LY6), and purple indicates that the favorable fragment is from Luoyang-9 (LY9). **b** Polymorphism analysis of the target genes among parents 9311, LYX, 1880, LY6 and LY9. The band genotype of *Bph9* in 9311 and 1880 was significantly lower than that of LY9, but slightly higher than that of LY6 and LYX. **c** Yield, quality and resistance of five parents. At the top is the morphology of mature panicles, in the middle is the performance of the milled grain, and at the bottom is the resistance of brown planthopper at seedling stage

The core of molecular design breeding is rational design, and our design strategy is shown as Additional file 1: Fig. S1. First, according to the requirements of rice production and the needs of the market, we set the goals to develop high yield, superior quality and high BPH-resistant rice. Then, we selected appropriate donors with the corresponding genes for high yield, superior quality and highly BPH resistance based on molecular markers (Fig. 1). The well-known, high-yielding, *indica* variety, 9311, containing the high-yield genes *APO1*, *Ghd7*, *Ghd8* and *Gn1a*, grain shape genes *GS3* and *qSW5*, and eating and cooking quality genes *Wx* and *ALK* (Additional file 1: Fig. S2), was used as the recurrent parent to further improve its yield, quality and BPH resistance. The recently released, high yielding variety, 1880, a CSSL carrying the large panicle gene *Gn8.1* from *O. longistaminata A.* in 9311 genetic background (Fig. 1c, Additional file 1: Fig. S2, Additional file 2: Table S1), was selected as the donor parent for the large panicle. The BPH-resistant lines, Luoyang-6 and Luoyang-9 are two CSSLs carrying BPH-resistant genes *Bph6* and *Bph9* in the 9311 background, respectively (Additional file 1: Fig. S2 and S3), were used as the donor parents for BPH resistance. Luo-Yu-Xiang, a superior quality rice derived from Basmati (Fig. 1c, Additional file 2: Table S2), was used as donor parent for the quality genes *SBE3*, *SSIV2*, *SSIII* and *GW7* (Additional file 1: Fig. S2). Finally, the dominant alleles *APO1*, *Ghd7*, *Ghd8*, *Gn1a*, *GS3*, *qSW5*, *Wx* and *ALK* in 9311, *Gn8.1* in 1880, *Bph6* in Luoyang-6, *Bph9* in Luoyang-9, *SBE3*, *SSIV2*, *SSIII* and *GW7* in Luo-Yu-Xiang, were selected as target genes for molecular design breeding and these five lines, 9311, 1880, Luoyang-6 (LY6), Luoyang-9 (LY9) and Luo-Yu-Xiang (LYX), were used as parents for pyramiding the desired target genes.

### Development of New Rice Lines with High-Yield, Superior-Quality and Highly BPH Resistance

**Fig. 2:**
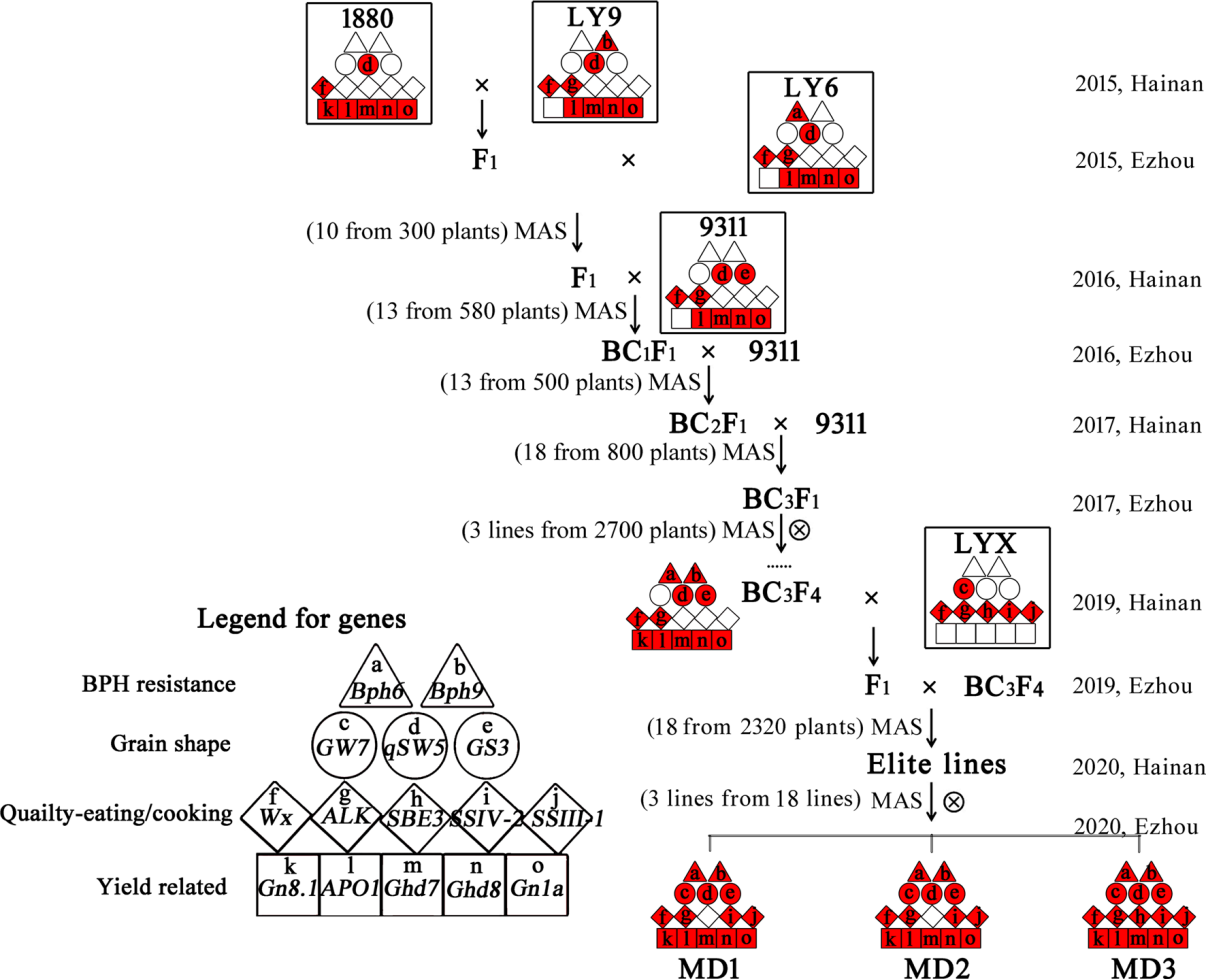
Schematic diagram of molecular design breeding process. The square represents yield-related genes, *Gn8.1* (k), *APO1* (l), *Ghd7* (m), *Ghd8* (n) and *Gn1a* (o); diamond represents eating and cooking quality related genes, *Wx* (f), *ALK* (g), *SBE3* (h), *SSIV-2* (i) and *SSIII-1* (j); circle represents grain shape related genes, *GW7* (c), *qSW5* (d) and *GS3* (e); triangle represents rice BPH resistance related genes, *Bph6* (a) and *Bph9* (b). The red fill represents the functional allelic fragments, and no color represents non-functional allelic fragments

After the parental rice lines were selected, pyramiding of the target genes was carried out by crossing the parents with each other as shown in Fig. 2. We first combined the excellent genes in 9311 (*APO1*, *Ghd7*, *Ghd8*, *Gn1a*, *Wx*, *ALK*, *GS3* and *qSW5*) with those in 1880, LY6 and LY9 which are CSSLs in the 9311 backgrounds. These four parents included 11 target genes (*Gn8.1*, *APO1*, *Ghd7*, *Ghd8*, *Gn1a*, *Wx*, *ALK*, *qSW5*, *GS3*, *Bph6* and *Bph9*), of which six, *APO1*, *Ghd7*, *Ghd8*, *Gn1a*, *Wx* and *qSW5*, from 9311 were included in all four parents (Additional file 1: Fig. S2), thus selection was only for *Gn8.1*, *ALK*, *GS3*, *Bph6* and *Bph9*. Foreground selection was performed from the F_1_ to BC_3_F_4_ generations and only progenies carrying all the target alleles were selected for backcrossing or selfing. In detail, from a F_1_ population about 300 lines derived from intercrossing the three parents (1880/LY9//LY6), ten F_1_ plants with all the target genes were identified. Then, three plants were selected based on background genotyping for further backcrossing as the maternal parent with 9311. In the BC_1_ generation, 13 out of 580 plants were identified as our expected candidates based on genotyping for the linked markers (Indel 33 for Gn8.1, RM16994 for Bph6 and RM28438 for Bph9) (Additional file 2: Table. S3) and phenotyping for the 9311 plant type with large panicles. Similarly, the selected BC_1_, BC_2_ plants were then backcrossed with 9311 to eliminate the unexpected background. Subsequently, 18 BC_3_F_1_ plants were selected for selfing until homozygous for targeted genes in BC_3_F_4_. Finally, three stable BC_3_F_4_ lines with target genes were developed from 2,700 plants (Fig. 2).

These three BC_3_F_4_ lines successfully pyramid all the 11 target genes (Additional file 1: Fig. S4), and exhibited the desired high yield and BPH resistance (Additional file 1: Fig. S5). Compared with 9311, the grain numbers per panicle of the three BC_3_F_4_ lines was increased by 51.2%, 65.4% and 52.5%, respectively, resulting in the plant yield increased by 43.1%, 60.7% and 46.6%, respectively (Additional file 2: Table S4). The resistance scores showed that the BPH resistance index of the three BC_3_F_4_ lines decreased from 9.0 in 9311 to 2.9, 3.0 and 3.1, respectively, meaning the plants exhibited high resistance (Additional file 1: Fig. S3). However, the quality of these lines showed no significant difference from that of 9311 (Additional file 2: Table S5), and needed improvement. For this purpose, a BC_3_F_4_ line was selected as the female parent for crossing with Luo-Yu-Xiang to introgress the desirable quality-related alleles, *SBE3*, *SSIV2*, *SSIII* and *GW7*. The F_1_ obtained from BC_3_F_4_/ Luo-Yu-Xiang was backcrossed with the same BC_3_F_4_ line, and the 2,320 backcrossed progenies were screened for the 15 targeted markers and desired phenotypes. From this evaluation, 32 superior lines were selected for selfing and 18 stable “molecular design breeding” lines were chosen for further evaluation. Of note, in order to obtain the breeding lines more quickly and pyramid of all 15 target genes, we retained some lines containing heterozygous fragments in the selected 32 superior lines. Subsequently, we selected for homozygosity at all 15 targeted genes in the process of selfing these lines and obtained 18 stable lines for further evaluation.

### Genetic Effects of Different Target Gene Combinations

**Fig. 3:**
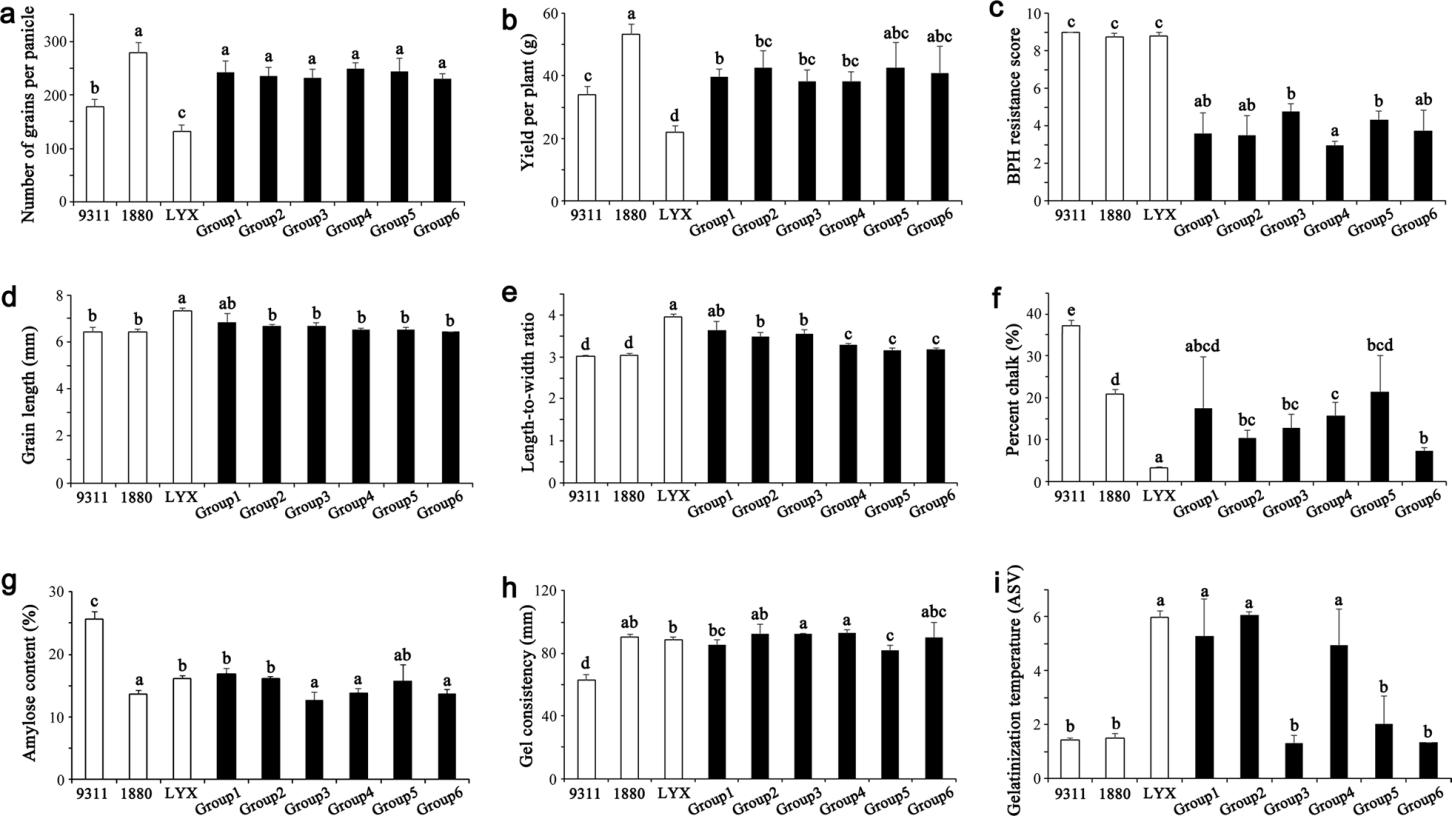
Phenotypic differences in groups with different target gene combinations. **a**: Number of grains per panicle; **b**: Yield per plant (g); **c**: BPH resistance; **d**: Grain length (mm); **e**: Length-to-width ratio; **f**: Percent chalk (%); **g**: Amylose content (%); **h**: Gel consistency (mm); **i**: Gelatinization temperature, which is represented by the alkali spreading value (ASV). Values are the means ± s.d.. Letters indicate a significant difference at the 5% significance level by the least significant difference test

In order to fully utilize the target genes in the molecular design breeding, we compared the genetic effects of different target gene combinations with the parent 9311 as the control. Based on combinations of target genes, the 18 molecular design breeding lines were divided into six groups (Additional file 1: Fig. S6). The five yield-related target genes, *Gn8.1*, *APO1*, *Ghd7*, *Ghd8* and *Gn1a*, were present in all six groups. These five genes controlled grain number, thus significantly increased the number of grains per panicle across all groups compared with 9311 (Fig. 3a). However, the increase of yield per plant was much smaller than grain number per panicle due to the differences in effective panicle number, spikelet fertility and 1000-grain weight among different groups (Fig. 3a, b; Additional file 1: Fig. S7). Similarly, the yield per plant varies greatly among different lines in the same group, requiring careful selection based on the comprehensive phenotypic performance (Additional file 2: Table S6). The six groups all harbored *Bph6* and *Bph9*, and the BPH resistance of the six groups was significantly improved relative to 9311 (Fig. 3c).

The differences between the groups were reflected in the set of quality-related genes *GW7*, *SSIV-2*, *SBE3* and *SSIII-1*. Group 1 included all the target quality genes, and the grain shape, parent chalk, amylose content, gel consistency and alkali spreading value were significantly improved compared with 9311 (Fig. 3). Group 2 did not include *SBE3* that regulates gel consistency and alkali spreading value (Tian et al. 2009), but the deletion of *SBE3* did not result in significant changes in gel consistency and alkali spreading value compared with group 1 (Fig. 3h, i; Additional file 1: Fig. S6), indicating little effect of *SBE3* on grain quality. Groups 3, 5 and 6 did not include *SSIV-2*, which regulates alkali spreading value (Tian et al. 2009), thus the alkali spreading value was consistent with 9311 as expected, and significantly lower than the superior parent Luo-Yu-Xiang (Fig. 3i, Additional file 1: Fig. S6), indicating the significant genetic effects of *SSIV-2* on alkali spreading value regulation. The deletion of *GW7*, a gene that controls the length-to-width ratio primarily by modulating grain width (Wang et al. 2015), resulted in significantly lower length-to-width ratio in Groups 4, 5 and 6 than the non-deletion Groups 1, 2 and 3 (Fig. 3e, Additional file 1: Fig. S6). Group 6 lost *SSIII-1* which regulates amylose content (Tian et al. 2009), however, the amylose content showed no significant difference among six groups (Fig. 3g), indicating the small genetic effect of *SSIII-1* on grain quality as that of *SBE3*.

In general, the target gene(s) showed obvious improvements to the corresponding target traits. However, in some cases, the grain yield and quality showed significant differences among lines in the same group, indicating the genetic complexity of grain yield and quality and some potential genes in the genetic background play important roles on these traits. For example, the Group1, lines 01, 13 and 14 contained all of the 15 target genes and the grain number per panicle was significantly increased by 44.9%, 41.6% and 22.5% compared with 9311, but the yield per plant was increased only by 22.9%, 10% and 18.2% due to lower seed setting rate and 1000-grain weight (Additional file 2: Table S6). The quality performance of the three lines was significantly improved compared to 9311. In detail, the ratio of length-to-width ratio, amylose content, gel consistency and alkali spreading value of line 01 were noticeably improved, but the percent chalk was still as high as 9311. For line 13, the percent chalk and alkali spreading value were not significantly improved relative to the superior parents Luo-Yu-Xiang. For line 14, the appearance quality and cooking quality were significantly improved, which were close to the level of superior-quality parent Luo-Yu-Xiang (Additional file 2: Table S7). Among these three lines, the performance of line 14 was particularly outstanding, and its yield, quality traits and resistance to brown planthopper were significantly improved. This indicates that careful phenotypic selection is still an indispensable auxiliary means for molecular design breeding.

In summary, the selected lines pyramided 14 or all 15 of the targeted genes, exhibiting improved BPH resistance, grain yield and rice quality relative to 9311. This improvement was especially apparent in lines 07, 08 and 14 which had superior performance compared to the donor parents and reached the target goals for yield, BPH resistance, grain shape, and quality. These three lines (07, 08 and 14) were renamed MD1 (Molecular design 1), MD2 and MD3, respectively.

### Genetic Background Profiling and Phenotyping of the Three MD Lines

**Fig. 4:**
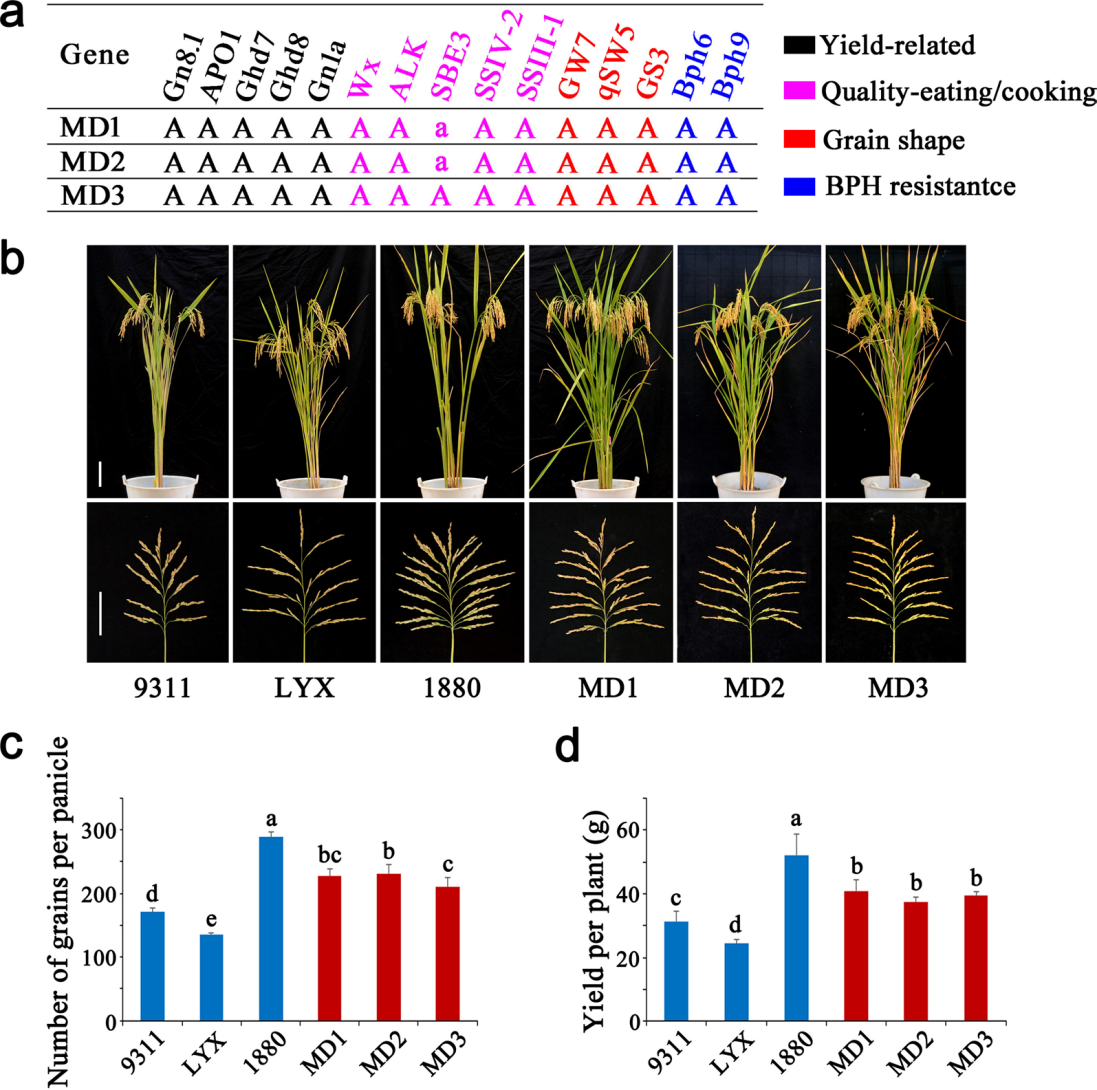
Gene distribution and yield performance of molecular design breeding lines. **a**: Genotype of the target genes in MD1, MD2 and MD3. Black represents yield-related genes, pink represents eating and cooking quality related genes, red represents appearance quality related genes, bule represents rice BPH resistance related genes. Uppercase letters represent functional alleles and lowercase letters represent nonfunctional alleles. **b**: Gross plant and panicle morphologies. Scale bars, 10 cm. **c**: Number of grains per panicle. **d**: Yield per plant. Values are the means ± s.d., n = 15. Letters indicate a significant difference at the 5% significance level by the least significant difference test

**Fig. 5:**
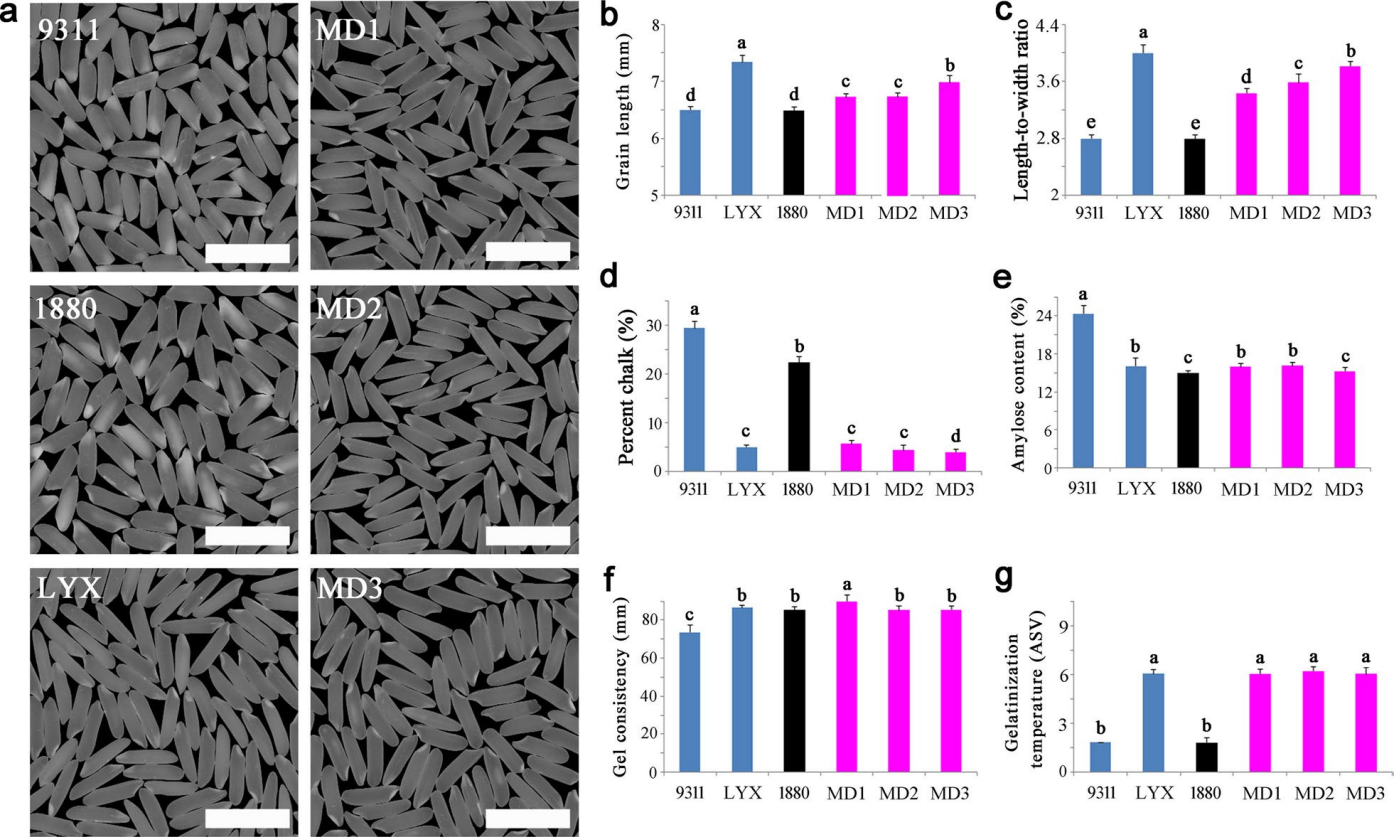
Rice quality performance of molecular design breeding lines and their parents. **a**: The performance of the milled grain. Scale bar, 1 cm. **b**: Grain length. **c**: Length-to-width ratio. **d**: Chalky grain rate. **e**: Amylose content. **f**: Gel consistency. **g**: Gelatinization temperature, which is represented by the alkali spreading value (ASV). Values are the means ± s.d., n = 15. Letters indicate a significant difference at the 5% significance level by the least significant difference test

**Fig. 6:**
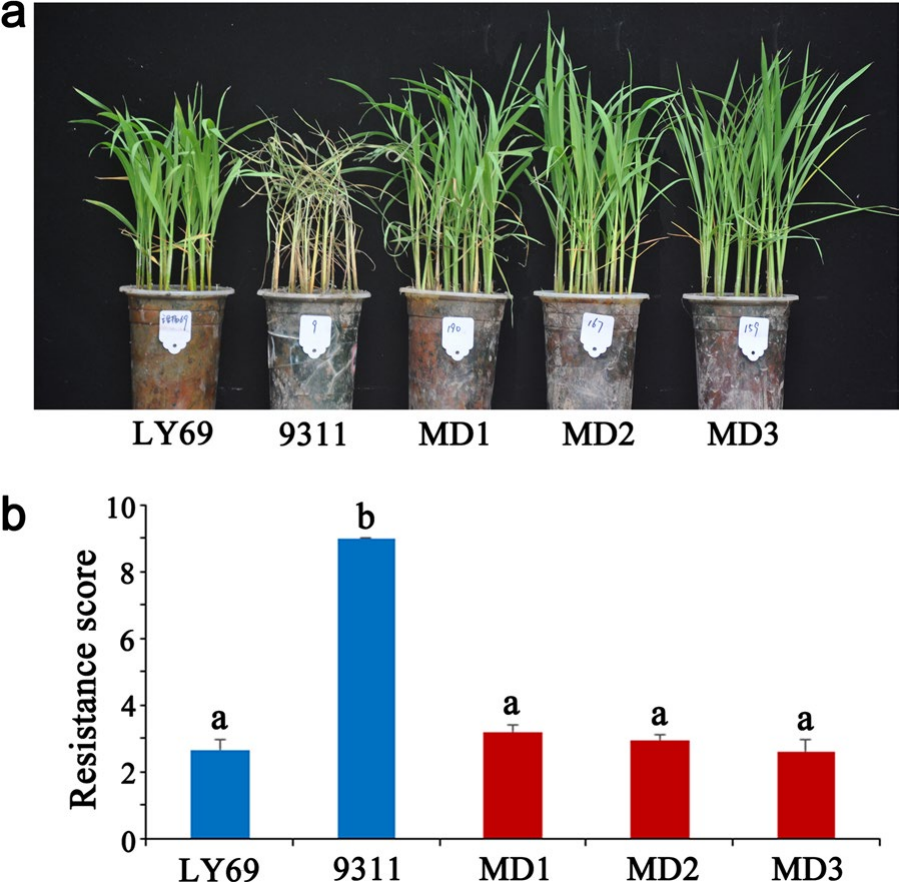
Resistance of brown planthopper in the molecular design breeding lines. **a**: Morphology of rice seedlings fed by brown planthopper. **b**: Brown planthopper resistance score. 9311 used as susceptible control, LY69 used as resistant control. Values are the means ± s.d., n = 30. Letters indicate a significant difference at the 5% significance level by the least significant difference test

Across the three selected elite rice lines, MD3 contained all 15 target genes, MD1 and MD2 harbored all target genes except *SBE3* (Fig. 4a). To determine whether the lines obtained the desired characters, we investigated their yield characters (Fig. 4b–d, Table 1), results showed that the MD1, MD2 and MD3 had excellent plant morphology, and inherited the characteristics of large panicle and high yield of 1880 (Fig. 4b), with the obvious yield advantages of the other donor parents. Relative to the Luo-Yu-Xiang, the grain yield of MD1, MD2 and MD3 increased about 56.8%, 47.6% and 55.9%, respectively; compared with the recurrent parent 9311, the grain yield increased about 21.7%, 14.6% and 21.0%, respectively (Table 1). Grain quality showed that the three lines had perfectly inherited the superior-quality character of Luo-Yu-Xiang, and the appearance quality and eating and cooking quality was noticeably improved (Fig. 5). The grain length and length-to-width ratio of the three lines were significantly higher than that of 9311, but slightly lower than that of Luo-Yu-Xiang. In terms of amylose content, gel consistency and alkali spreading value, the three lines were all significantly improved compared with 9311, and reached the high-quality level of the superior-quality parent Luo-Yu-Xiang. Also, for the percent chalk, the three lines were significantly improved and showed low chalkiness like Luo-Yu-Xiang (Fig. 5, Table 2). Brown planthopper resistance evaluations determined the resistance scores of MD1, MD2 and MD3 were 3.2, 2.9 and 2.6, respectively, which was similar to LY69 (2.7) (Fig. 6), exhibiting high resistance.

**Table 1:** Agronomic traits of selected molecular design lines and their parents in the design breeding

Lines	DTH	PH (cm)	PN	NGP	SF (%)	GW (g)	YP (g)	GY (kg/plot)
9311	90.0 ± 1.0^a^	123 ± 6^ab^	7.0 ± 1.0^b^	171 ± 6^d^	90.2 ± 0.8^a^	29.2 ± 0.2^a^	31.3 ± 3.3^c^	2.95 ± 0.37^c^
1880	86.8 ± 1.5^b^	126 ± 5^ab^	7.0 ± 1.0^b^	288 ± 8^a^	88.6 ± 1.3^ab^	29.4 ± 0.4^a^	52.1 ± 6.5^a^	4.56 ± 0.66^a^
LYX	89.5 ± 1.2^a^	120 ± 4^b^	8.2 ± 0.3^a^	135 ± 3^e^	87.3 ± 1.8^b^	25.2 ± 0.4^c^	24.4 ± 1.3^d^	2.29 ± 0.16^d^
LY6	90.5 ± 1.7^a^	125 ± 7^ab^	7.1 ± 1.5^a^	173 ± 10^d^	86.8 ± 1.7^b^	29.1 ± 0.4^a^	31.0 ± 2.3^c^	2.89 ± 0.43^c^
LY9	90.2 ± 1.9^a^	125 ± 8^ab^	7.2 ± 1.3^a^	175 ± 15^d^	84.8 ± 1.4^c^	29.3 ± 0.7^a^	30.8 ± 2.8^c^	2.85 ± 0.67^c^
MD1	88.9 ± 1.9^ab^	127 ± 8^a^	7.3 ± 0.6^b^	227 ± 12^bc^	88.3 ± 1.1^ab^	27.7 ± 0.7^b^	40.9 ± 3.4^b^	3.79 ± 0.40^b^
MD2	89.1 ± 2.1^ab^	126 ± 2^a^	7.4 ± 0.8^b^	231 ± 14^b^	89.1 ± 1.3^ab^	24.6 ± 1.1^c^	37.6 ± 1.3^b^	3.51 ± 0.26^b^
MD3	88.3 ± 1.7^ab^	123 ± 7^ab^	8.1 ± 0.2^a^	210 ± 15^c^	88.4 ± 2.7^ab^	26.1 ± 0.7^bc^	39.4 ± 1.5^b^	3.67 ± 0.28^b^

*DTH* day to heading; *PH* plant height; *PN* panicle number; *NGP* number of grains per panicle; *SF* spikelet fertility; *GW* 1000-grain weight; *YP* yield per plant; Values are the means ± s.d., n = 15. *GY* Grain yield per plot; means ± s.d., n = 3. Letters indicate a significant difference at the 5% significance level by the least significant difference test

**Table 2:** The grain quality characters of parents and three molecular design breeding lines

Materials	GL (cm)	L/W	PC (%)	AC (%)	GC (mm)	ASV (GT)
9311	6.38 ± 0.12^d^	3.04 ± 0.04^e^	28.3 ± 0.87^a^	24.5 ± 0.73^a^	73.3 ± 1.32^c^	1.50 ± 0.00^c^
1880	6.37 ± 0.11^d^	2.99 ± 0.04^e^	22.0 ± 0.92^b^	15.1 ± 0.39^c^	85.5 ± 1.37^b^	1.57 ± 0.17^c^
LYX	7.13 ± 0.17^a^	3.98 ± 0.06^a^	4.7 ± 0.35^c^	16.5 ± 0.33^b^	86.7 ± 1.51^b^	6.03 ± 0.32^a^
LY6	6.41 ± 0.15^d^	3.05 ± 0.06^e^	28.5 ± 0.88^a^	23.9 ± 0.83^a^	85.3 ± 1.47^b^	2.51 ± 0.41^b^
LY9	6.45 ± 0.12^d^	3.03 ± 0.07^e^	27.9 ± 0.51^a^	24.5 ± 1.57^a^	85.1 ± 3.85^b^	2.97 ± 0.52^b^
MD1	6.65 ± 0.12^c^	3.41 ± 0.09^d^	4.6 ± 0.22^c^	16.7 ± 0.39^b^	90.3 ± 1.35^a^	6.02 ± 0.28^a^
MD2	6.70 ± 0.13^c^	3.63 ± 0.05^c^	4.3 ± 0.25^c^	17.1 ± 0.42^b^	85.5 ± 1.27^b^	6.17 ± 0.33^a^
MD3	6.93 ± 0.16^b^	3.85 ± 0.06^b^	3.5 ± 0.18^d^	15.7 ± 0.45^c^	85.3 ± 1.37^b^	6.01 ± 0.39^a^

*GL* Grain length; *L*/*W* Length-to-width ratio; *PC* Percent chalk; *AC* Amylose content; *GC* Gel consistency; *ASV* Alkali spreading value. Values are the means ± s.d., n = 15. Letters indicate a significant difference at the 5% significance level by the least significant difference test

Finally, in order to further identify the genetic background of the three lines, 258 polymorphic SSR markers were selected that were evenly distributed across all 12 chromosomes (Additional file 2: Table S8). The lines MD1, MD2 and MD3 shared 85.0%, 82.5% and 82.4% of the genetic background with 9311, respectively (Additional file 2: Table S9). The remaining background came from the other four donor parents, 1880, Luoyang 6, Luoyang 9 and especially Luo-Yu-Xiang where MD1, MD2 and MD3 harbored its 23, 27 and 26 segments, respectively (Additional file 1: Fig. S9). This is consistent with the fact that most of the genes for quality improvement came from Luo-Yu-Xiang.

## Discussion

The ultimate goal of rice breeding is to create super-varieties by pyramiding multiple desirable traits, such as high yield, superior quality, insect resistance and environmental stress tolerance into one variety. In order to achieve this goal, breeders have made many attempts and realized some achievements in the transition from traditional breeding to marker-assisted selection breeding and most recently, by molecular design breeding (Kumar et al. 2016; Liu et al. 2016; Fan et al. 2017; Zeng et al. 2017). Molecular design breeding is a concept proposed on the basis of in-depth understanding of gene regulatory networks for complex traits, which is characterized by the integration of multiple genes and traits through rational design of multiple regulatory networks for complex traits (Peleman and van der Voort 2003; Zeng et al. 2017). Therefore, molecular design breeding has unique advantages in realizing multi-trait combinations just as verified in our study that the three complex traits of high-yield, superior-quality and high BPH resistance were quickly and effectively combined together by this method.

In molecular design breeding, selection and balance among yield, quality and resistance is the key to achieving rapid collection of multiple complex traits through rational design (Qian et al. 2016; Wang et al. 2020). Previous studies suggested that yield was the most complex trait, and it would be more efficient if we first introduce the rice quality or resistance-related traits into high-yielding germplasm in rice breeding programs (Wang et al. 2017; Zeng et al. 2017). In this study, the BPH-resistant gene was first introduced into 1880 (with a 9311 background), and then crossed with Luo-Yu-Xiang to improve rice quality, which is consistent with the strategy above. Although we successfully obtained three excellent lines through molecular design breeding, there are still many uncertainties in the selection process. When yield, resistance and rice quality related genes are clustered together, the agronomic traits of the lines become more complex. In terms of rice yield, the number of grains per panicle was significantly increased in the selected lines with all yield-related genes, but the yield per plant varied significantly among the lines (Additional file 2: Table S6). This is because incorporating the high quality parental genetic background affects the 1000-grain weight, seed-setting rate and panicle number, which subsequently affects the yield per plant. In terms of rice quality, the 18 selected lines which divided into six groups based on the 15 targeted genes, all showed significantly lower amylose content than 9311 (Fig. 3g), even though, the six groups carried the same *Wx* allele as 9311 (Additional file 1: Fig. S7). This phenomenon cannot be explained solely from the perspective of the *Wx* gene. Interestingly, we determined both 1880 and 9311 carried the *Wx*^*b*^ haplotype, but the amylose content of 1880 was significantly lower than that of 9311 (Fig. 3g, Additional file 1: Fig. S2). This suggests that 1880 may contain a potentially unknown gene that reduces amylose content independent of *Wx*, and it may be linked to the *Gn8.1*, leading to the linkage inheritance of lower amylose content and large panicle in the selected lines. These phenomena indicate that determining how to break the linkage between favorable alleles and unexpected genes will be a critical challenge in future molecular design breeding.

## Conclusions

We have successfully created three novel rice lines with high yield, superior quality and significantly improved BPH resistance by rational molecular design. These newly bred rice lines exhibited higher yield potential, better grain quality and higher BPH resistance than their parents. Our results demonstrate that molecular design is a powerful strategy to improve multiple complex traits and provides a reference for the future commercial rice improvement.

## Materials and Methods

### Plant Materials

9311, a well-known high-yielding *indica* variety, was developed in China in 1997. To date, 9311 has been used as a parent for developing over 20 varieties and is not only an excellent conventional rice variety, but also an excellent restorer line (https://www.ricedata.cn/). 9311 contains the high-yield genes *APO1*, *Ghd7*, *Ghd8* and *Gn1a*, grain shape genes *GS3* and *qSW5*, and eating and cooking quality genes *Wx* and *ALK* (Additional file 1: Fig. S2). These excellent genes make 9311 have high commercial value, so it has become a classic material for breeding improvement (Fan et al. 2017; Wang et al. 2017). 9311 is freely available and can be obtained from Professor Hongxi Zhang, Institute of Agriculture of Yangzhou City, Jiangsu Province, China.

1880, a chromosome segment substitution line (CSSL) in the background of 9311 which has the large panicle gene *Gn8.1* introgressed from *O. longistaminata A.* (International Rice Genebank Collection (IRGC) 101,211) on Chr. 8 at 25 Mb. 1880 is a new excellent high yield variety, with 68.4% more grains per panicle and 54.5% higher yield compared to 9311 (Fan et al. 2017). This variety can be obtained from Professor Shaoqing Li, College of Life Science, Wuhan University in the future when it is made publicly available.

Luoyang-6 and Luoyang-9, are CSSLs in the 9311 backgrounds, carrying BPH-resistant genes *Bph6* and *Bph9*, respectively. The BPH resistance was introgressed from the *indica* variety Swarnalata with *Bph6* on Chr. 4 at 23 Mb (Qiu et al. 2010; Guo et al. 2018) and Pokkali (IRGC 108921) with *Bph9* on Chr.12 at 22 Mb (Zhao et al. 2016). These two CSSLs were provided by Professor Guangcun He, College of Life Science, Wuhan University and can be obtained directly from Professor He.

Luo-Yu-Xiang, a superior quality rice derived from Basmati (IRIS_313-11567). Luo-Yu-Xiang contains a large number of dominant alleles for quality-related genes *SBE3*, *SSIV2*, *SSIII* and *GW7* that 9311 does not possess, and also contains quality related genes *Wx* and *ALK* that 9311 does possess (Additional file 1: Fig. S2). These excellent genes endowed Luo-Yu-Xiang with superior quality characteristics, such as low chalkiness, low amylose content, high gel consistency and high alkali spreading value. China's rice consumption market is mainly *indica* rice, and the slender type of *indica* rice is favored by consumers. Therefore, the slender grain characteristics of Luo-Yu-Xiang is also regarded as the target trait of combination breeding. This variety can be obtained from Professor Shaoqing Li, College of Life Science, Wuhan University in the future when it is made publicly available.

The development of the selected lines is described in the Results section “Development of new rice lines with high-yield, superior-quality and BPH resistance” and Fig. 2.

### Field Experiment

The five parents (9311, 1880, Luoyang-6, Luoyang-9 and Luo-Yu-Xiang) and the 18 “elite lines” (Fig. 2) which include three selected lines MD1, MD2 and MD3 were planted in a rice paddy fields at the experimental field of Wuhan University in Ezhou (114°35′ E, 30°10′ N) in summer (sowing on May 20th) and Hainan (110°02′ E, 18°48′ N) in winter (sowing on November 20th) during 2015–2020. All seeds were soaked in water at 37 °C in the dark for 24 h and were germinated at 37 °C for 12 h. The 25-day-old seedlings were transplanted in a ten-row plots with 10 plants per row with 20 cm × 26 cm spacing. Three plots were planted for each line as three replications and 100 plants were planted in each plot. All plots were planted in a randomized complete block design. Standard field management was adopted with medium fertility level. Similar cultivation practices were performed each year.

### Investigation of Agronomic Traits in the Field

The measurements of the day to heading (DTH), plant height (PH), panicle number (PN), number of grains per panicle (NGP), spikelet fertility (SF), 1000-grain weight (TGW) and yield per plant (YP) were performed at 30 days after heading. For all these traits, five representative plants in the middle of each plot were chosen for trait measurement. As a result, a total of 15 individual plants were used to evaluate the agronomic traits of each line. The statistical analyses were performed with SPSS Statistics 20 (IBM, USA).

### Investigation of the Grain Quality

One hundred fully filled dehulled grains were randomly selected to determine grain length, grain width, length-to-width ratio and chalky kernels using the Wanshen SC-E type rice appearance quality detection analyzer (Hangzhou, China). Percent chalk is defined as the percentage of all rice grains that contain chalkiness. The amylose content (AC), gel consistency (GC) and alkali spreading value (ASV) were measured as previously described (Tian et al. 2009). Each trait was measured with three replications.

### Evaluation of Resistance to Brown Planthopper in Rice Materials

The resistance of brown planthopper (BPH) in rice populations was evaluated by seedling identification using the previously reported method (Huang et al. 2001; Fan et al. 2017). Luoyang 69 (LY69), a line which has both the *Bph6* and *Bph9* genes with high BPH resistance (Wang et al. 2017), was used as the resistant control, while 9311 was the susceptible control. When all of the 9311 seedlings died, the other lines were examined and each seedling was given a score of 1 to 9 according to the method of Huang et al. (2001). The test was replicated three times and the average of the three replications was the resistance score.

### Target Gene Screening and Background Profiling by Molecular Marker Analysis

Fifteen genes including *Wx*, *ALK*, *SBE3*, *SSIV2*, *SSIII*, *GW7*, *GS3*, *qSW5*, *Gn8.1*, *APO1*, *Ghd7*, *Ghd8*, *Gn1a*, *Bph6* and *Bph9*, were selected as target genes and selected by foreground selection in each generation of intercrossing and/or backcrossing (Fig. 2) using gene-specific PCR (Additional file 2: Table S3). A total of 672 pairs of SSR primers evenly distributed across the 12 chromosomes were screened as potential background markers. From this screening, 258 SSR markers showed polymorphic differences among the five parents, thus these SSRs were selected for genetic background screening (Additional file 2: Table S8). The physical map was drawn using MapMaker Version 3.0 (Lander et al. 1987). The percentages of chromosome segments from recurrent parent in molecular design lines were calculated as previously described (Xi et al. 2006; Suh et al. 2013).

## Supplementary Information

**Additional file 1: Fig. S1** Flow chart of breeding by molecular design; **Fig. S2** Distribution of the target genes in selected parent lines. Black represents yield-related genes, pink represents eating and cooking quality related genes, red represents grain shape related genes, blue represents rice BPH resistance related genes. Uppercase letters represent functional alleles and lowercase letters represent nonfunctional alleles. (Gene description in Table S8); **Fig. S3** BPH resistance score of the parents and BC_3_F_4_ lines at the seedling stage. 9311 used as susceptible control, LY69 used as resistant control. Values are the means ± s.d., n = 30. Letters indicate a significant difference at the 5% significance level by the least significant difference test; **Fig. S4** Genotyping of the target genes in BC_3_F_4_ lines. Black represents yield-related genes, pink represents eating and cooking quality related genes, red represents grain shape related genes, blue represents rice BPH resistance related genes. Uppercase letters represent functional alleles and lowercase letters represent nonfunctional alleles. Lines 07, 08 and 14 were subsequently selected and renamed MD1, MD2 and MD3, respectively. (Gene description in Table S8.); **Fig. S5** Gross plant and panicle morphologies of the BC_3_F_4_ lines and their parents. Scale bars, 10 cm; **Fig. S6** Genotypes of the 18 selected lines based on the 15 target genes. Black represents yield-related genes, pink represents eating and cooking quality related genes, red represents grain shape related genes, blue represents rice BPH resistance related genes. Uppercase letters represent functional alleles and lowercase letters represent nonfunctional alleles. (Phenotypic data in Tables S5 and S6. Gene description in Table S8); **Fig. S7** Yield-related traits in groups with different target gene combinations. **a**: Plant height (cm); **b**: Panicle number; **c**: Spikelet fertility (%); **d**: 1000-grian weight (g). Values are the means ± s.d.. Letters indicate a significant difference at the 5% significance level by the least significant difference test; **Fig. S8** BPH resistance test of the designed breeding lines at the seedling stage. Values are the means ± s.d., n = 30. Letters indicate a significant difference at the 5% significance level by the least significant difference test; **Fig. S9** Target genes and genetic background analysis of the molecular designed breeding lines. Letters A, B and C represent the molecular design breeding lines MD1, MD2 and MD3, respectively. The box color in parenthesis indicates the substituted chromosome segments of the donor parents 1880 (black), Luo-Yu-Xiang, LYX (green), Luoyang-6, LY6 (red) and Luoyang-9, LY9 (blue) with 9311 as the recurrent background (white).

**Additional file 2: Table S1** Agronomic trait performance of the parents in designed breeding; **Table S2** The grain quality characters of the parents in designed breeding; **Table S3** Target genes and related markers used in this study; **Table S4** Agronomic trait performance of the BC_3_F_4_ lines and their parents; **Table S5** The grain quality characters of the BC_3_F_4_ lines and their parents; **Table S6** Agronomic trait performance of the parents and 18 elite lines. Lines 07, 08 and 14 were subsequently selected and renamed MD1, MD2 and MD3, respectively. (Target genes for Lines 01 to 18 in Fig. S6.); **Table S7** The grain quality characters of the parents and 18 elite lines. Lines 07, 08 and 14 were subsequently selected and renamed MD1, MD2 and MD3, respectively. (Target genes for Lines 01 to 18 in Fig. S6.); **Table S8** 258 SSR markers used in this study; **Table S9** Genetic background screening information of the design breeding lines.

## Data Availability

All data generated or analyzed during this study are included in this published article and its additional files.

## Abbreviations

BPH: Brown planthopper
MAS: Marker-assisted selection
QTL: Quantitative trait loci
BIL: Backcross inbred line
LYX: Luo-Yu-Xiang
LY6: Luoyang-6
LY9: Luoyang-9
MD: Molecular design
PH: Plant height
PN: Panicle number
NGP: Number of grains per panicle
SF: Spikelet fertility
TGW: 1000-Grain weight
YP: Yield per plant
AC: Amylose content
GC: Gel consistency
ASV: Alkali spreading value
