# Supplementary material for: Creation of Elite Rice with High-Yield, Superior-Quality and High Resistance to Brown Planthopper Based on Molecular Design
Source: Rice (N Y). 2022 Mar 15;15:17. doi: 10.1186/s12284-022-00563-7 (PMC8924342; doi:10.1186/s12284-022-00563-7)
Supplement: Supplementary file 1 — Additional file 1: Fig. S1 Flow chart of breeding by molecular design; Fig. S2 Distribution of the target genes in selected parent lines. Black represents yield-related genes, pink represents eating and cooking quality related genes, red represents grain shape related genes, blue represents rice BPH resistance related genes. Uppercase letters represent functional alleles and lowercase letters represent nonfunctional alleles. (Gene description in Table S8); Fig. S3 BPH resistance score of the parents and BC3F4 lines at the seedling stage. 9311 used as susceptible control, LY69 used as resistant control. Values are the means ± s.d., n = 30. Letters indicate a significant difference at the 5% significance level by the least significant difference test; Fig. S4 Genotyping of the target genes in BC3F4 lines. Black represents yield-related genes, pink represents eating and cooking quality related genes, red represents grain shape related genes, blue represents rice BPH resistance related genes. Uppercase letters represent functional alleles and lowercase letters represent nonfunctional alleles. Lines 07, 08 and 14 were subsequently selected and renamed MD1, MD2 and MD3, respectively. (Gene description in Table S8.); Fig. S5 Gross plant and panicle morphologies of the BC3F4 lines and their parents. Scale bars, 10 cm; Fig. S6 Genotypes of the 18 selected lines based on the 15 target genes. Black represents yield-related genes, pink represents eating and cooking quality related genes, red represents grain shape related genes, blue represents rice BPH resistance related genes. Uppercase letters represent functional alleles and lowercase letters represent nonfunctional alleles. (Phenotypic data in Tables S5 and S6. Gene description in Table S8); Fig. S7 Yield-related traits in groups with different target gene combinations. a: Plant height (cm); b: Panicle number; c: Spikelet fertility (%); d: 1000-grian weight (g). Values are the means ± s.d.. Letters indicate a significant d [file 12284_2022_563_MOESM1_ESM.docx]

**Creation of elite rice with high-yield, superior-quality and high resistance to brown planthopper based on molecular design**

Manman Liu^†^, Fengfeng Fan^†^, Shihao He, Yu Guo, Gaili Chen, Nannan Li, Nengwu Li, Huanran Yuan, Fengfeng Si, Fang Yang, Shaoqing Li*

State Key Laboratory of Hybrid Rice, Hongshan Laboratory of Hubei Province, Key Laboratory for Research and Utilization of Heterosis in Indica Rice of Ministry of Agriculture, Engineering Research Center for Plant Biotechnology and Germplasm Utilization of Ministry of Education, College of Life Science, Wuhan University, Wuhan 430072, China.

*Corresponding author:

Shaoqing Li

Email: shaoqingli@whu.edu.cn

^†^Manman Liu and Fengfeng Fan contributed equally to this work.

**Supplementary materials**

**
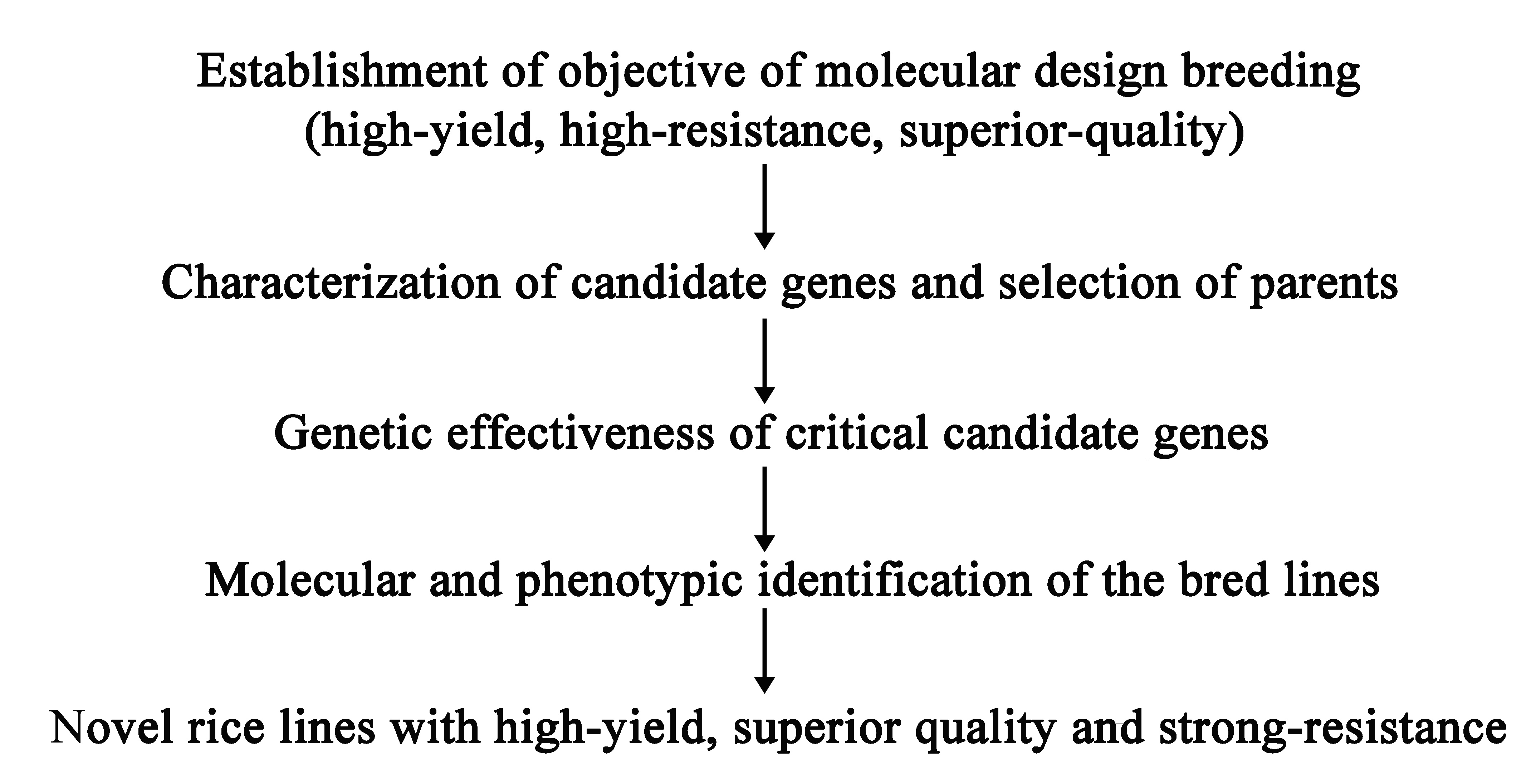
**

**Fig. S1** **Flow chart of breeding by molecular design.**


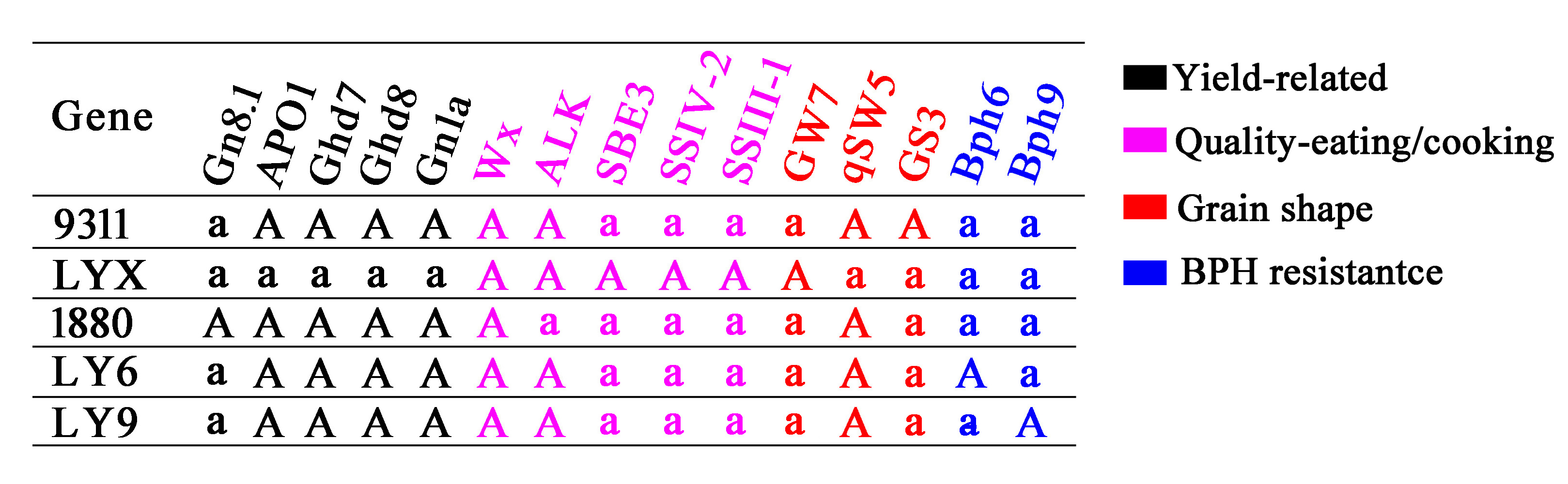


**Fig. S2** **Distribution of the target genes in selected parent lines.** Black represents yield-related genes, pink represents eating and cooking quality related genes, red represents grain shape related genes, blue represents rice BPH resistance related genes. Uppercase letters represent functional alleles and lowercase letters represent nonfunctional alleles. (Gene description in Table S8.)


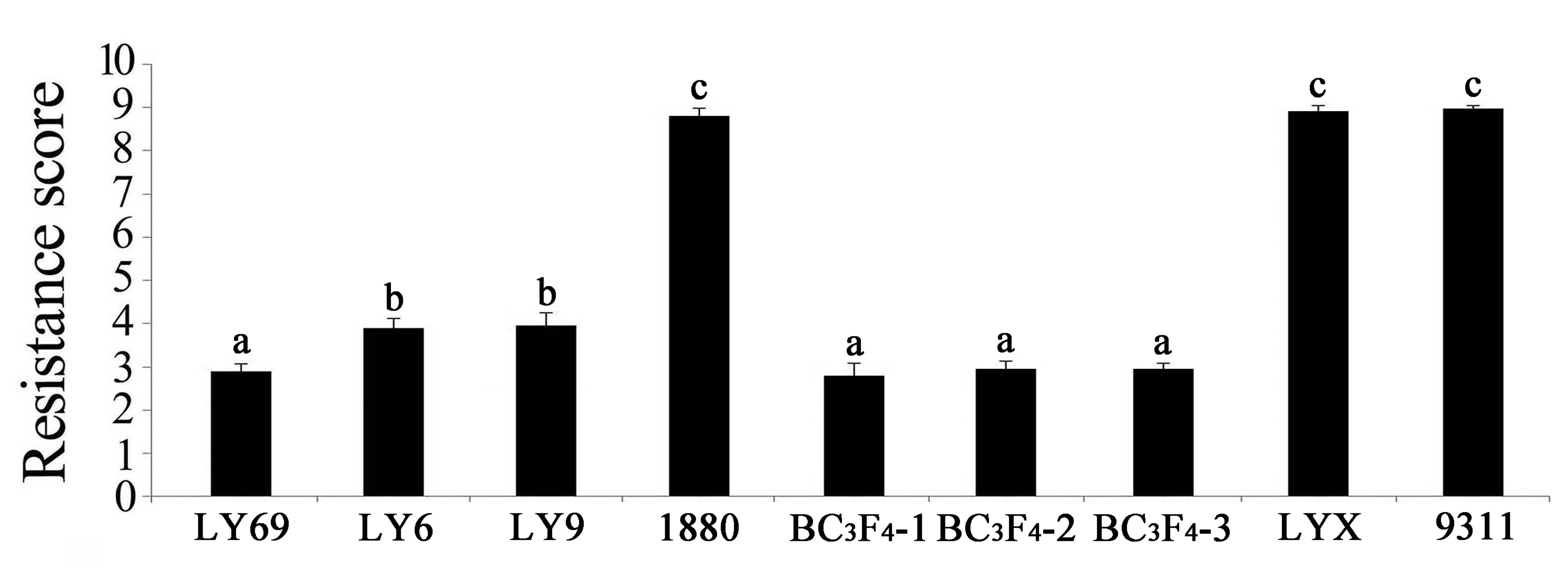


**Fig. S3** **BPH resistance score of the parents and BC_3_F_4_ lines at the seedling stage.**  9311 used as susceptible control, LY69 used as resistant control. Values are the means ± s.d., n=30. Letters indicate a significant difference at the 5% significance level by the least significant difference test.


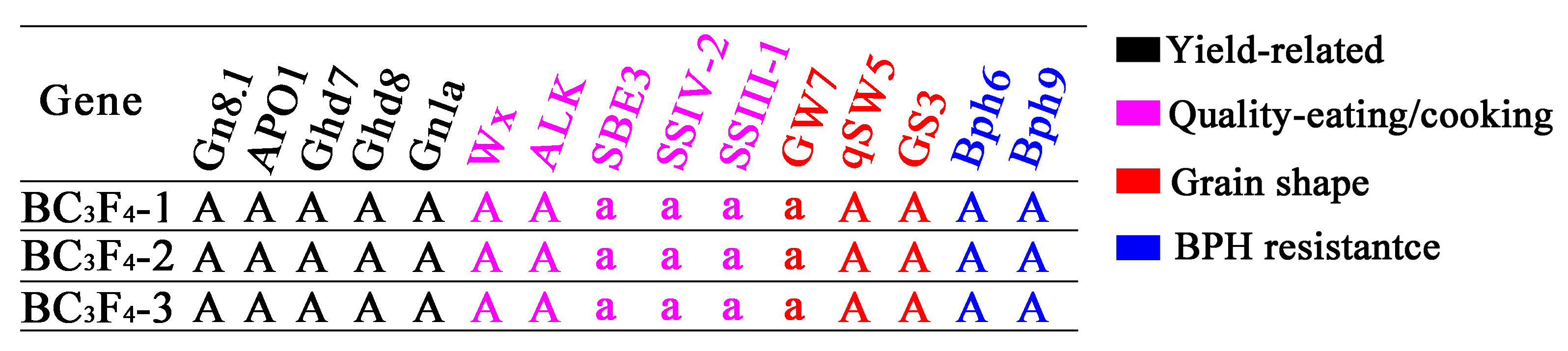


**Fig. S4** **Genotyping of the target genes in BC_3_F_4_ lines.** Black represents yield-related genes, pink represents eating and cooking quality related genes, red represents grain shape related genes, blue represents rice BPH resistance related genes. Uppercase letters represent functional alleles and lowercase letters represent nonfunctional alleles. (Gene description in Table S8.)


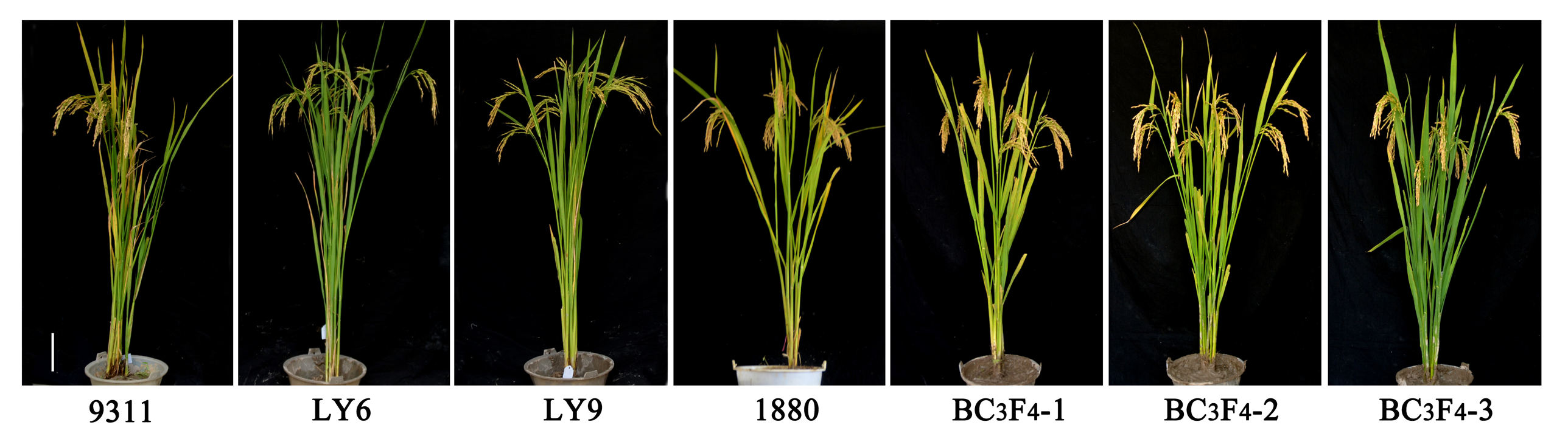


**Fig. S5** **Gross plant and panicle morphologies of the BC_3_F_4_ lines and their parents**. Scale bars, 10 cm.


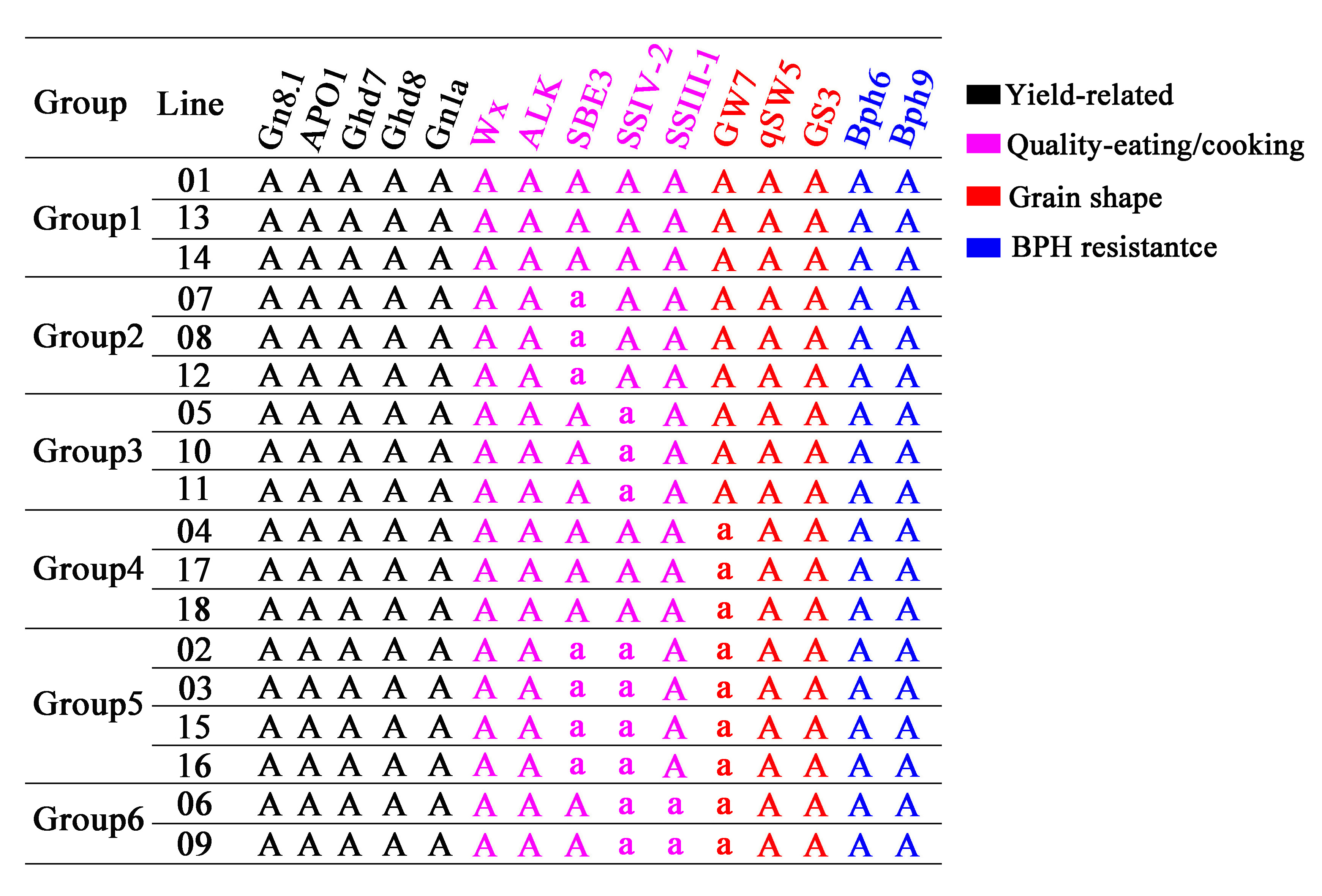


**Fig. S6** **Genotypes of the 18 selected lines based on the 15 target genes.** Black represents yield-related genes, pink represents eating and cooking quality related genes, red represents grain shape related genes, blue represents rice BPH resistance related genes. Uppercase letters represent functional alleles and lowercase letters represent nonfunctional alleles. Lines 07, 08 and 14 were subsequently selected and renamed MD1, MD2 and MD3, respectively. (Phenotypic data in Tables S5 and S6. Gene description in Table S8.)

**
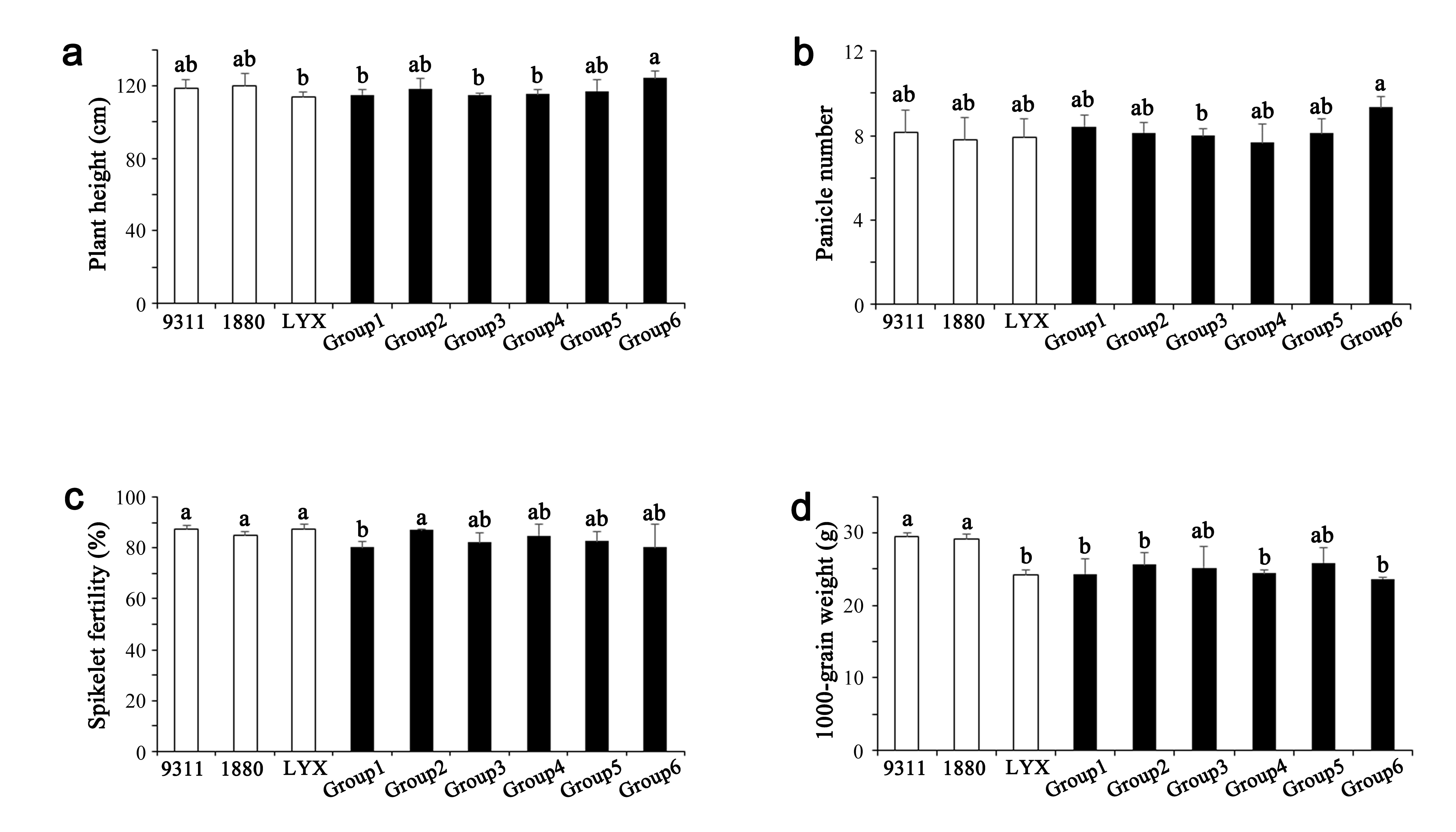
**

**Fig. S7 Yield-related traits in groups with different target gene combinations.** **a**: Plant height (cm); **b**: Panicle number; **c**: Spikelet fertility (%); **d**: 1000-grian weight (g). Values are the means ± s.d.. Letters indicate a significant difference at the 5% significance level by the least significant difference test.


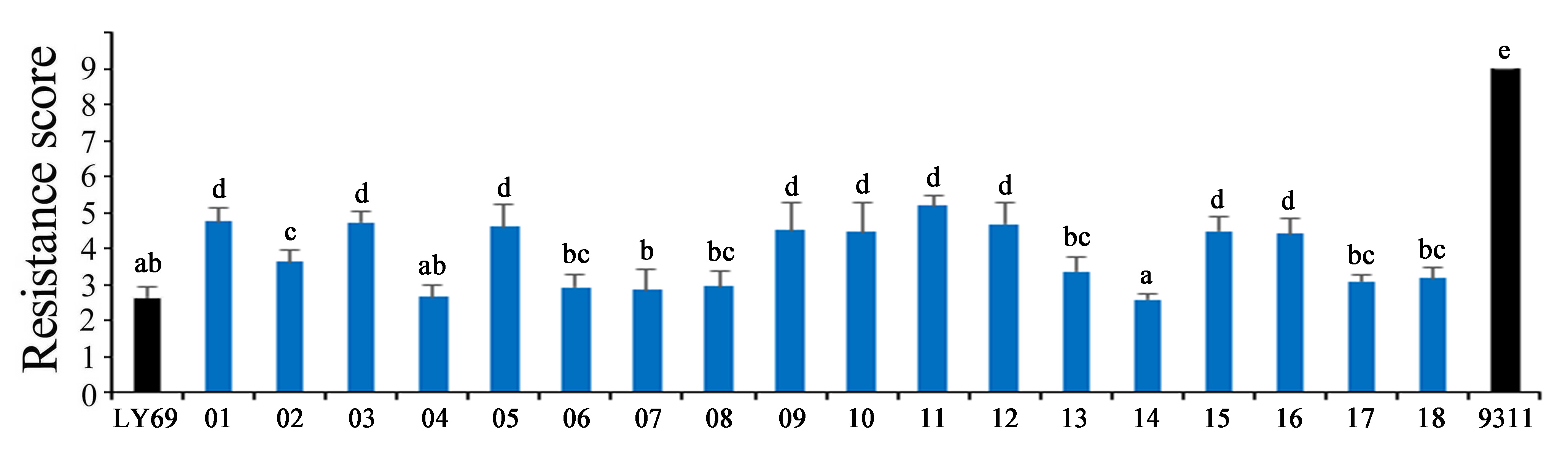


**Fig. S8** **BPH resistance test of the designed breeding lines at the seedling stage.** Values are the means ± s.d., n=30. Letters indicate a significant difference at the 5% significance level by the least significant difference test.


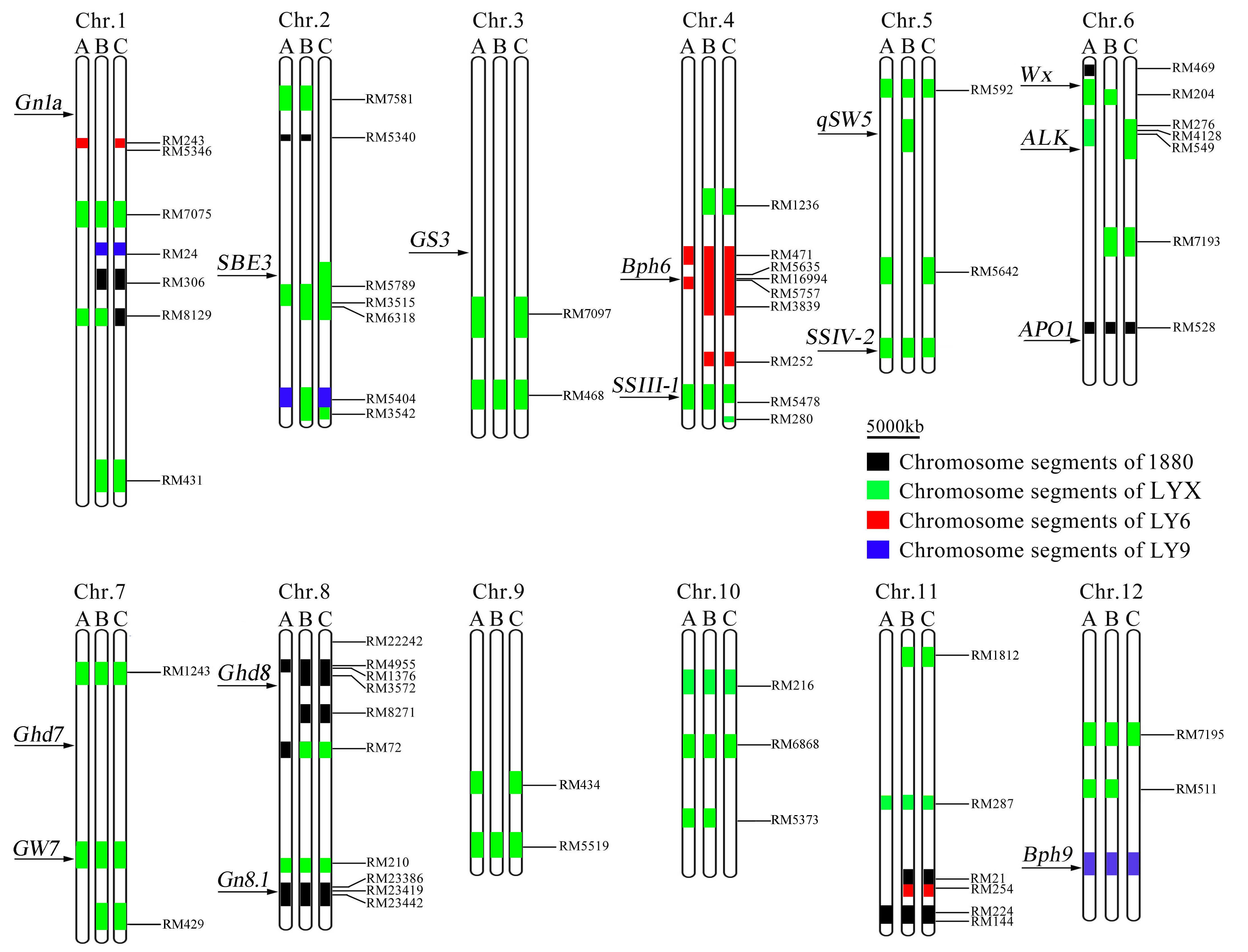


**Fig. S9** **Target genes and genetic background analysis of the molecular designed breeding lines.** Letters A, B and C represent the molecular design breeding lines MD1, MD2 and MD3, respectively. The box color in parenthesis indicates the substituted chromosome segments of the donor parents 1880 (black), Luo-Yu-Xiang, LYX (green), Luoyang-6, LY6 (red) and Luoyang-9, LY9 (blue) with 9311 as the recurrent background (white).
