# Supplementary material for: Creation of Elite Rice with High-Yield, Superior-Quality and High Resistance to Brown Planthopper Based on Molecular Design
Source: Rice (N Y). 2022 Mar 15;15:17. doi: 10.1186/s12284-022-00563-7 (PMC8924342; doi:10.1186/s12284-022-00563-7)
Supplement: Supplementary file 2 — Additional file 2: Table S1 Agronomic trait performance of the parents in designed breeding; Table S2 The grain quality characters of the parents in designed breeding; Table S3 Target genes and related markers used in this study; Table S4 Agronomic trait performance of the BC3F4 lines and their parents; Table S5 The grain quality characters of the BC3F4 lines and their parents; Table S6 Agronomic trait performance of the parents and 18 elite lines. Lines 07, 08 and 14 were subsequently selected and renamed MD1, MD2 and MD3, respectively. (Target genes for Lines 01 to 18 in Fig. S6.); Table S7 The grain quality characters of the parents and 18 elite lines. Lines 07, 08 and 14 were subsequently selected and renamed MD1, MD2 and MD3, respectively. (Target genes for Lines 01 to 18 in Fig. S6.); Table S8 258 SSR markers used in this study; Table S9 Genetic background screening information of the design breeding lines. [file 12284_2022_563_MOESM2_ESM.docx]

**Creation of elite rice with high-yield, superior-quality and high resistance to brown planthopper based on molecular design**

Manman Liu^†^, Fengfeng Fan^†^, Shihao He, Yu Guo, Gaili Chen, Nannan Li, Nengwu Li, Huanran Yuan, Fengfeng Si, Fang Yang, Shaoqing Li*

State Key Laboratory of Hybrid Rice, Hongshan Laboratory of Hubei Province, Key Laboratory for Research and Utilization of Heterosis in Indica Rice of Ministry of Agriculture, Engineering Research Center for Plant Biotechnology and Germplasm Utilization of Ministry of Education, College of Life Science, Wuhan University, Wuhan 430072, China.

*Corresponding author:

Shaoqing Li

Email: shaoqingli@whu.edu.cn

^†^Manman Liu and Fengfeng Fan contributed equally to this work.

**Table S1 Agronomic traits performance of the parents in designed breeding.**

| Materials | PH (cm) | PN | NGP | SF (%) | TGW (g) | YP (g) |
| --- | --- | --- | --- | --- | --- | --- |
| 9311 | 121±7 | 8.0±1.3 | 177±18 | 88.3±2.1 | 29.4±0.4 | 36.7±2.2 |
| 1880 | 122±6 | 7.5±1.5 | 298±27 | 87.2±1.5 | 29.1±0.5 | 56.7±2.8 |
| LYX | 113±5 | 7.5±0.5 | 135±12 | 89.3±1.3 | 24.1±0.5 | 21.8±1.7 |
| LY6 | 126±5 | 7.9±1.5 | 175±13 | 86.1±1.5 | 29.3±0.5 | 34.9±1.9 |
| LY9 | 125±6 | 8.2±1.3 | 170±17 | 83.8±1.7 | 29.5±0.6 | 34.5±2.8 |

Note：PH, Plant height; PN, panicle number; NGP, number of grains per panicle; SF, spikelet fertility; TGW, 1000-grain weight; YP, yield per plant; Values are the means ± s.d., n=15.

**Table S2 The grain quality characters of the parents in designed breeding.**

| Materials | GL (cm) | L/W | PC (%) | AC (%) | GC (mm) | ASV (GT) |
| --- | --- | --- | --- | --- | --- | --- |
| 9311 | 6.43±0.17 | 3.07±0.04 | 28.3±1.72 | 24.3±1.51 | 73.5±2.75 | 2.60±0.22 |
| 1880 | 6.39±0.15 | 3.03±0.03 | 22.1±1.55 | 14.7±0.69 | 87.1±1.62 | 2.30±0.27 |
| LYX | 7.28±0.15 | 3.95±0.03 | 2.8±0.53 | 17.1±0.47 | 92.3±1.87 | 6.12±0.29 |
| LY6 | 6.51±0.09 | 3.08±0.07 | 28.1±0.45 | 24.7±0.55 | 83.5±2.44 | 3.53±0.38 |
| LY9 | 6.42±0.15 | 3.01±0.08 | 27.2±0.29 | 25.2±2.51 | 82.5±3.71 | 3.91±0.22 |

Note：GL, Grain length; L/W, Length-to-width ratio; PC, Percent chalk; AC, Amylose content; GC, Gel consistency; ASV, Alkali spreading value; Values are the means ± s.d., n=15.

**Table S3 Target genes and related markers used in this study.**

| **Gene** | **Name** | **Linked marker** | **Primer sequences** | **Temperature** | **Reference** |
| --- | --- | --- | --- | --- | --- |
| *Wx* | *Waxy* gene | Wx-1 | F: ACCATTCCTTCAGTTCTTTGTCT | 55 | Zeng et al. 2017 |
|  |  |  | R: CTGATGAACAACAGAACAACACA |  |  |
| *ALK* | *Alkali degeneration* gene | ALK-1 | F: TAGCGACTATGGTTTGTGTGTG | 55 | Zeng et al. 2017 |
|  |  |  | R: ACTCCAGTCCAGCCGTCAG |  |  |
| *SBE3* | *Starch branching enzyme 3* | SBE3-1 | F: GAATCAACCATCCAGCAAAG | 55 | Zeng et al. 2017 |
|  |  |  | R: GATAAGTTGTTACAAGTTTCCTCTGT |  |  |
| *SSIV-2* | *Starch synthase-IV-2* | SSIV-2-1 | F: GTTTGGGTTGGGTTGGGTT | 55 | Zeng et al. 2017 |
|  |  |  | R: ACGCACCACCAAGGGAATT |  |  |
| *SSIII-1* | *Starch synthase-III-1* | SSIII-1 | F: AAGAAGGGAAGGGAGTCAGC | 55 | Tian et al. 2009 |
|  |  |  | R: GCCATCTCCATTGCCAGC |  |  |
| *GW7* | *Grain width 7* | Gw7-1 | F: ATTAGCTGCAACTCGTTCTGC | 55 | Wang et al. 2015 |
|  |  |  | R: TGGAAGAGTATGGCCTAGAAA |  |  |
| *GS3* | *Grain size 3* | GS3-1 | F: TAGCCGCCTCTGGCTGGAT | 55 | Zeng et al. 2017 |
|  |  |  | R: TTCCACATCTCCCCTCCCCT |  |  |
| *qSW5* | QTL for *seed width 5* | qSW5-1 | F: AGTACGACCATGATGTTTCCC | 55 | Zeng et al. 2017 |
|  |  |  | R: GACCTAACCCATCTCATTCCA |  |  |
| *Gn8.1* | *Grain number 8.1* | Indel33 | F: GCAAGAACTCGTGCCAAACAT | 55 | unpublished data |
|  |  |  | R: ACTGTTCTATTGCCTATTGGGGA |  |  |
| *APO1* | *Aberrant panicle organization 1* | Apo1-1 | F: TTGGTTTGTCTCAGCTCTGATCT | 55 | Zeng et al. 2017 |
|  |  |  | R: GAAATGATGAACACTGTCCAACA |  |  |
| *Ghd7* | *Grain number, plant height and heading date 7* | Ghd7-1 | F: TATCCTATGTATCCCAGCACCAT | 55 | Zeng et al. 2017 |
|  |  |  | R: CCATTCAGAAAACTTGATTGCAT |  |  |
| *Ghd8* | *Grain number, plant height and heading date 8* | Ghd8-1 | F: TCTATCAAGGTGCTCTATCGCT | 55 | Zeng et al. 2017 |
|  |  |  | R: ACATGTAATGCAAACAACCAAGT |  |  |
| *Gn1a* | *Grain number 1a* | Gn1a-1 | F: TTTCCAGAGTATTTTTATGGTTCT | 55 | Zeng et al. 2017 |
|  |  |  | R: ATGTTACTGTCCCACCTGAAAC |  |  |
| *Bph6* | *Brown planthopper resistant 6* | RM16994 | F: TGGCAGTACACACTACAGTACATGC | 55 | Guo et al. 2018 |
|  |  |  | R: AGAGGGAGGAGAGAAAGGAAGG |  |  |
| *Bph9* | *Brown planthopper resistant 9* | RM28438 | F: GTTCGTGAGCCACAACAAATCC | 55 | Zhao et al. 2016 |
|  |  |  | R: GTTAAATGCTCCACCAAACACACC |  |  |

**Table S4** **Agronomic trait performance of the BC_3_F_4_ lines and their parents.**

| Materials | PH (cm) | PN | NGP | SF (%) | TGW (g) | YP (g) |
| --- | --- | --- | --- | --- | --- | --- |
| 9311 | 103±5 | 8.2±1.1 | 162±22 | 87.5±1.8 | 29.2±0.5 | 33.9±2.5 |
| 1880 | 105±6 | 7.7±1.4 | 260±28 | 86.3±1.9 | 29.0±0.3 | 50.1±1.9 |
| LY6 | 110±7 | 7.5±1.5 | 175±15 | 85.8±1.7 | 29.5±0.7 | 33.2±1.7 |
| LY9 | 111±6 | 7.8±1.5 | 173±13 | 86.3±1.5 | 29.1±0.3 | 33.9±1.8 |
| BC_3_F_4_-1 | 105±6 | 7.8±1.3 | 245±15 | 85.1±1.5 | 29.8±0.6 | 48.5±1.7 |
| BC_3_F_4_-2 | 106±7 | 7.9±1.3 | 268±27 | 85.5±1.5 | 30.1±0.6 | 54.5±1.9 |
| BC_3_F_4_-3 | 105±5 | 7.7±1.2 | 247±22 | 84.3±1.8 | 29.7±0.5 | 49.7±2.7 |

Note：PH, Plant height; PN, panicle number; NGP, number of grains per panicle; SF, spikelet fertility; TGW, 1000-grain weight; YP, yield per plant; Values are the means ± s.d., n=15.

**Table S5** **The grain quality characters of the BC_3_F_4_ lines and their parents.**

| Materials | GL (cm) | L/W | PC (%) | AC (%) | GC (mm) | ASV (GT) |
| --- | --- | --- | --- | --- | --- | --- |
| 9311 | 6.44±0.12 | 3.09±0.08 | 30.5±0.52 | 23.7±1.12 | 75.5±1.89 | 2.51±0.28 |
| 1880 | 6.41±0.13 | 3.06±0.06 | 28.6±0.39 | 15.5±0.83 | 88.3±1.77 | 1.72±0.31 |
| LY6 | 6.38±0.05 | 3.05±0.04 | 29.3±0.58 | 23.3±0.72 | 80.5±1.33 | 2.68±0.59 |
| LY9 | 6.41±0.11 | 3.03±0.06 | 28.3±0.33 | 23.9±0.77 | 81.3±2.70 | 2.73±0.13 |
| BC_3_F_4_-1 | 6.41±0.09 | 3.03±0.11 | 25.3±0.15 | 17.5±0.25 | 88.3±1.53 | 2.82±0.19 |
| BC_3_F_4_-2 | 6.51±0.13 | 3.09±0.15 | 25.9±0.31 | 17.9±1.37 | 85.3±2.13 | 2.78±0.73 |
| BC_3_F_4_-3 | 6.43±0.31 | 3.11±0.08 | 26.2±0.32 | 16.5±1.62 | 86.6±1.45 | 2.63±0.52 |

Note：GL, Grain length; L/W, Length-to-width ratio; PC, Percent chalk; AC, Amylose content; GC, Gel consistency; ASV, Alkali spreading value; Values are the means ± s.d., n=15.

**Table S6** **Agronomic trait performance of the parents and 18 elite lines.** Lines 07, 08 and 14 were subsequently selected and renamed MD1, MD2 and MD3, respectively. (Target genes for Lines 01 to 18 in Fig. S6.)

| Group | Materials | PH (cm) | PN | NGP | SF (%) | TGW (g) | YP (g) |
| --- | --- | --- | --- | --- | --- | --- | --- |
|  | 9311 | 119±5 | 8.2±1.1 | 178±15 | 87.5±1.8 | 29.5±0.5 | 34.0±2.5 |
|  | 1880 | 120±7 | 7.7±1.7 | 278±20 | 85.1±1.3 | 29.1±0.7 | 53.1±3.2 |
|  | LYX | 114±3 | 7.9±0.9 | 132±13 | 87.1±1.9 | 24.3±0.7 | 22.1±2.1 |
| Group 1 | 01 | 118±5 | 9.0±1.0 | 258±4 | 78.2±2.4 | 23.1±0.6 | 41.8±4.6 |
|  | 13 | 112±5 | 8.0±1.0 | 252±15 | 80.4±3.6 | 23.2±0.5 | 37.4±5.4 |
|  | 14 | 115±5 | 8.3±1.0 | 218±21 | 82.7±7.3 | 26.8±0.4 | 40.2±5.8 |
| Group 2 | 07 | 114±4 | 8.7±1.2 | 231±13 | 87.8±4.2 | 27.5±1.0 | 47.7±1.8 |
|  | 08 | 125±6 | 7.7±1.2 | 223±26 | 86.8±4.0 | 25.0±0.7 | 36.9±3.8 |
|  | 12 | 117±5 | 8.0±1.7 | 254±23 | 86.7±3.1 | 24.3±0.9 | 43.0±4.9 |
| Group 3 | 05 | 116±5 | 8.0±1.7 | 238±7 | 80.4±2.0 | 22.4±1.2 | 34.2±6.7 |
|  | 10 | 114±7 | 7.7±0.9 | 244±44 | 79.5±4.8 | 28.4±1.8 | 42.0±4.2 |
|  | 11 | 115±3 | 8.3±0.6 | 213±13 | 86.7±3.3 | 24.7±0.5 | 38.1±3.4 |
| Group 4 | 04 | 113±7 | 6.5±1.2 | 262±40 | 89.1±2.3 | 24.3±0.6 | 35.3±2.4 |
|  | 17 | 116±3 | 8.0±0.9 | 245±38 | 85.4±1.9 | 24.9±0.5 | 41.7±2.8 |
|  | 18 | 118±6 | 8.0±1.1 | 241±42 | 80.3±2.1 | 24.2±0.3 | 37.4±2.9 |
| Group 5 | 02 | 120±4 | 8.7±0.6 | 245±20 | 83.1±5.2 | 26.7±1.0 | 47.1±5.1 |
|  | 03 | 112±5 | 8.7±1.5 | 230±20 | 87.8±2.3 | 28.4±0.6 | 50.2±10.9 |
|  | 15 | 112±3 | 7.7±1.2 | 223±21 | 78.9±1.3 | 23.6±0.5 | 31.8±5.0 |
|  | 16 | 125±3 | 7.3±0.6 | 278±18 | 81.4±4.1 | 24.8±0.3 | 41.4±5.1 |
| Group 6 | 06 | 122±7 | 9.0±1.0 | 223±16 | 74.0±3.3 | 23.5±1.3 | 34.8±2.9 |
|  | 09 | 127±6 | 9.7±1.0 | 237±12 | 86.8±0.6 | 23.8±2.4 | 46.9±4.0 |

Note：PH, Plant height; PN, panicle number; NGP, number of grains per panicle; SF, spikelet fertility; TGW, 1000-grain weight; YP, yield per plant; Values are the means ± s.d., n=15.

**Table S7** **The grain quality characters of the parents and 18 elite lines.** Lines 07, 08 and 14 were subsequently selected and renamed MD1, MD2 and MD3, respectively. (Target genes for Lines 01 to 18 in Fig. S6.)

| Group | Materials | GL (cm) | L/W | PC (%) | AC (%) | GC (mm) | ASV(GT) |
| --- | --- | --- | --- | --- | --- | --- | --- |
|  | 9311 | 6.41±0.22 | 3.01±0.03 | 37.4±1.15 | 25.6±1.21 | 62.3±3.71 | 1.50±0.00 |
|  | 1880 | 6.43±0.12 | 3.03±0.05 | 20.1±1.12 | 13.6±0.59 | 90.7±1.58 | 1.50±0.17 |
|  | LYX | 7.33±0.11 | 3.96±0.05 | 3.2±0.23 | 16.2±0.37 | 88.7±1.97 | 5.83±0.32 |
| Group 1 | 01 | 6.53±0.31 | 3.40±0.21 | 28.1±0.25 | 17.7±0.55 | 86.3±2.35 | 6.12±0.28 |
|  | 13 | 6.73±0.23 | 3.67±0.22 | 20.3±0.21 | 17.0±1.47 | 81.5±4.29 | 3.68±0.27 |
|  | 14 | 7.23±0.35 | 3.83±0.21 | 4.2±0.49 | 15.8±1.10 | 88.2±12.40 | 6.01±0.83 |
| Group 2 | 07 | 6.64±0.21 | 3.45±0.13 | 8.2±1.28 | 15.7±1.21 | 93.7±5.59 | 6.00±0.00 |
|  | 08 | 6.67±0.24 | 3.60±0.18 | 10.6±0.34 | 16.3±1.78 | 98.0±1.73 | 6.05±0.12 |
|  | 12 | 6.74±0.22 | 3.41±0.17 | 12.2±0.25 | 16.3±0.55 | 85.2±2.35 | 6.20±0.28 |
| Group 3 | 05 | 6.56±0.22 | 3.64±0.11 | 13.3±1.59 | 13.5±1.38 | 93.0±2.77 | 1.00±0.33 |
|  | 10 | 6.85±0.25 | 3.47±0.12 | 9.3±0.52 | 13.2±3.84 | 92.7±6.04 | 1.54±0.17 |
|  | 11 | 6.62±0.25 | 3.57±0.16 | 15.6±2.15 | 11.2±3.47 | 92.3±4.40 | 1.39±0.99 |
| Group 4 | 04 | 6.49±0.42 | 3.25±0.23 | 13.4±2.15 | 13.3±1.50 | 95.0±5.65 | 3.35±0.98 |
|  | 17 | 6.57±0.29 | 3.32±0.15 | 19.3±1.45 | 13.9±2.10 | 90.8±4.37 | 5.85±0.77 |
|  | 18 | 6.52±0.36 | 3.29±0.18 | 14.2±1.87 | 14.5±2.10 | 93.2±4.65 | 5.62±0.42 |
| Group 5 | 02 | 6.44±0.26 | 3.22±0.17 | 23.6±0.21 | 17.2±2.47 | 83.5±4.29 | 2.87±0.33 |
|  | 03 | 6.64±0.41 | 3.16±0.12 | 8.8±0.12 | 16.0±3.60 | 85.3±2.54 | 2.96±0.69 |
|  | 15 | 6.58±0.22 | 3.17±0.13 | 27.6±0.72 | 17.7±2.16 | 80.4±9.15 | 1.21±0.68 |
|  | 16 | 6.47±0.24 | 3.11±0.22 | 25.8±0.59 | 12.1±3.95 | 78.4±9.26 | 1.06±0.66 |
| Group 6 | 06 | 6.44±0.19 | 3.16±0.09 | 7.9±4.18 | 13.2±2.69 | 97.0±2.23 | 1.32±0.25 |
|  | 09 | 6.43±0.21 | 3.21±0.09 | 6.8±0.75 | 14.2±1.53 | 83.3±1.67 | 1.33±0.14 |

Note：GL, Grain length; L/W, Length-to-width ratio; PC, Percent chalk; AC, Amylose content; GC, Gel consistency; ASV, Alkali spreading value; Values are the means ± s.d., n=15.

**Table S8 258 SSR markers used in this study.**

| Markers | Chromosome number | Forward Primer | Reverse Primer |
| --- | --- | --- | --- |
| RM24 | 1 | CTAAATTTCTGGCCGTAGGATCTTGG | GGGTAGTGGACGGCGAATGC |
| RM226 | 1 | GAAGCTAAGGTCTGGGAGAAACC | AATGGCCTTAACCAAGTAGGATGG |
| RM243 | 1 | CAGACTGCAGTTGCACGATACTACG | GAAAGCTGCAACGATGTTGTCC |
| RM246 | 1 | CGAGCTCCATCAGCCATTCAGC | ACTTGAGAGCGAGATTGGGAATCG |
| RM297 | 1 | ACAGGGCTATGCAGACACAGTGC | AGCAAGCGAAGGGAAGTGACC |
| RM430 | 1 | GTCCCTGATCAGAAACGAGATGG | TAGGGTTGGAAGAATGCAAGACC |
| RM472 | 1 | CATTGACGTGGCACTTTGTTCC | AGAGAGCACGCAATGGAGTATGC |
| RM510 | 1 | GTTTGACGCGATAAACCGACAGC | ATGAGGACGACGAGCAGATTCC |
| RM562 | 1 | GGAAAGGAAGAATCAGACACAGAGC | GTACCGTTCCTTTCGTCACTTCC |
| RM572 | 1 | CGCGGTTAATGTCATCTGATTGG | CCATACTTCGAGATCCAAGACTGACC |
| RM575 | 1 | GTAGCCATAGCCTTTCCCAAAGC | TCTCCTGCAGCTGATTTCTTGG |
| RM576 | 1 | GCAGTAATATGTGGAGGTTTCG | GTAGGTCAAAGCCTTCTATCAGC |
| RM578 | 1 | AGATATACACGGCAATCCGATCC | GTAGGGTTTGAAAGCTTGAAGTGC |
| RM579 | 1 | TTCCGAGTGGTTATGCAAATGG | TCGTGACCTGAGAAATTGTGTCC |
| RM581 | 1 | ATGCGTGATCAACAATCGAGAACG | CCATAGCCGATGGATTGAAAGTGG |
| RM583 | 1 | GTTGCGGTTTGTTCGTTCTTGC | TAGATCCCAGCAGACGGATCAGC |
| RM1361 | 1 | ATGCTTGCAGACAATCGATGC | CTCTCCGCCTAAACAACTTGTGC |
| RM1843 | 1 | GACTGATGGTCACTTGCAGTTCG | CTTTCCAAGCACAGCCTAAGTGG |
| RM3148 | 1 | GCTTTGGTATTTGCAGGTTCACG | CTATTGCTCGAACACTTTGCTTCTCC |
| RM5346 | 1 | GCTACTCCACAGCGTTAGCTTCG | GACGGAGGAGCCGAACTTGG |
| RM6324 | 1 | CTGTACAAGAACGGCAGCAACC | GCACCACCAAACAGAGACAGAGG |
| RM7075 | 1 | GCGTTGCAGCGGAATTTGTAGG | CCCTGCTTCTCTCGTGCAGTCG |
| RM8004 | 1 | TCTCTACCACCGTCTTCTCTCTCC | CTGAACGCAGCCACATCTAAGC |
| RM8129 | 1 | CTCAACCCGGCTTTCCATCTCG | GCTGCAGAGTCTCGCACGTTCC |
| RM8126 | 1 | TGGGCCTCTTTGTTTCATACTCC | TCCTCATCTCTCTCCGTGTCTCC |
| RM154 | 2 | GACGGTGACGCACTTTATGAACC | CGATCTGCGAGAAACCCTCTCC |
| RM180 | 2 | CCTTCTCCTTCTTTCAGCTTCTGC | CAACTTGCTCTACTTGTGGTGAGG |
| RM208 | 2 | AGTACCACCACCATTCTCTGCAAGC | TCGATTGGCCATGAGTTCTCG |
| RM240 | 2 | CCTTAATGGGTAGTGTGCAC | TGTAACCATTCCTTCCATCC |
| RM279 | 2 | GCGGGAGAGGGATCTCCT | GGCTAGGAGTTAACCTCGCG |
| RM341 | 2 | CAAGAAACCTCAATCCGAGC | CTCCTCCCGATCCCAATC |
| RM475 | 2 | CCTCACGATTTTCCTCCAAC | ACGGTGGGATTAGACTGTGC |
| RM482 | 2 | TCTGAAAGCCTGACTCATCG | GTCAATTGCAGTGCCCTTTC |
| RM561 | 2 | GAGCTGTTTTGGACTACGGC | GAGTAGCTTTCTCCCACCCC |
| RM2265 | 2 | AACTGACCGTATATTAGCCA | TGACCGCCTCTATTATATTG |
| RM3515 | 2 | ACGCTTGTGGTGTTTAATAC | CACTGTGAATACACAGGAAC |
| RM3542 | 2 | CTCCATGGAAAGCTAGCCAG | AATCACCCTTTCAGTGCCTC |
| RM5340 | 2 | TGGGCCCTAAGTCATATTGC | ACCCAACGAGATGTACCTCG |
| RM5345 | 2 | CAGATACCCTCGCAAAAAGG | CGGACCCCAAAGAAAGAAAG |
| RM5346 | 2 | TGCCTCACGATGGTCGAG | CTTCGTCCACCCAATTTGAC |
| RM5404 | 2 | GGCCATCCATCTCCTGTATG | GACACACACAGGGTTGGTTG |
| RM5699 | 2 | ATCGTTTCGCATATGTTT | ATCGGTAAAAGATGAGCC |
| RM5789 | 2 | CAGCTCACCGCATCACAC | GGACGCCATCATGGTGAG |
| RM6318 | 2 | TGCTGCTTCTGTCCAGTGAG | GGATCATAACAAGTGCCTCG |
| RM7581 | 2 | CATTTCAACTAGTAAGCGTGTC | TTACAGCCGCTATGATAAGG |
| RM132 | 3 | ATCTTGTTGTTTCGGCGGCGGC | CATGGCGAGAATGCCCACGTCC |
| RM168 | 3 | TGCTGCTTGCCTGCTTCCTTT | GAAACGAATCAATCCACGGC |
| RM203 | 3 | CCTATCCCATTAGCCAAACATTGC | GATTTACCTCGACGCCAACCTG |
| RM251 | 3 | GAATGGCAATGGCGCTAG | ATGCGGTTCAAGATTCGATC |
| RM448 | 3 | TCTGATCTTGATGCAGGCAC | TCTCCCGATTTGGACAGATC |
| RM468 | 3 | CCCTTCCTTGTTGTGGCTAC | TGATTTCTGAGAGCCAACCC |
| RM514 | 3 | AGATTGATCTCCCATTCCCC | CACGAGCATATTACTAGTGG |
| RM520 | 3 | AGGAGCAAGAAAAGTTCCCC | GCCAATGTGTGACGCAATAG |
| RM571 | 3 | GGAGGTGAAAGCGAATCATG | CCTGCTGCTCTTTCATCAGC |
| RM1278 | 3 | CCATAGCAATTTAGCCATAT | TCTAATTCTCCCCAACACTA |
| RM1319 | 3 | GTGCTAAGCTTCTTCTGTGC | GCCAGTTAGCCCTTAAATC |
| RM3392 | 3 | GTCCAATGATTCGTTCCCAC | CTTCACCGTTCACCAATTCC |
| RM3436 | 3 | GCATCCCGGTGACTAGTACG | TGTGCATGTGGTAAGGAACC |
| RM3867 | 3 | TTGACTGGAACATCGAGCTC | ATCCCCTCTACACCGTACCC |
| RM5626 | 3 | GATCAGTCGGTCATAAACG | CACCTTCCTCTTCTGCTG |
| RM6929 | 3 | GTTGAACATATTGTGGGGGC | GGTTCTGGACTACATCGAACG |
| RM7097 | 3 | GGGAGGAGGAGAGGAGATTG | TTAGGCCTGCACTTTTGGAG |
| RM8204 | 3 | TTTGGCATTTCTACCATTTC | TTTGCAATGACCAAATATAGG |
| RM119 | 4 | CATCCCCCTGCTGCTGCTGCTG | CGCCGGATGTGTGGGACTAGCG |
| RM142 | 4 | CTCGCTATCGCCATCGCCATCG | TCGAGCCATCGCTGGATGGAGG |
| RM177 | 4 | CCCTCTTAGACAGAGGCCAGAGGG | GTAGCCGAAGATGAGGCCGCCG |
| RM185 | 4 | AGTTGTTGGGAGGGAGAAAGGCC | AGGAGGCGACGGCGATGTCCTC |
| RM252 | 4 | TTCGCTGACGTGATAGGTTG | ATGACTTGATCCCGAGAACG |
| RM255 | 4 | TGCCCATATGGTCTGGATG | GAAAGTGGATCAGGAAGGC |
| RM280 | 4 | GTGCTCTCCATGTCGGATTATGC | CAAGGCAACAAGATTGGTTAGTGG |
| RM303 | 4 | GCATGGCCAAATATTAAAGG | GGTTGGAAATAGAAGTTCGGT |
| RM335 | 4 | GTACACACCCACATCGAGAAG | GCTCTATGCGAGTATCCATGG |
| RM401 | 4 | TGGAACAGATAGGGTGTAAGGG | CCGTTCACAACACTATACAAGC |
| RM417 | 4 | CGGATCCAAGAAACAGCAG | TTCGGTATCCTCCACACCTC |
| RM471 | 4 | ACGCACAAGCAGATGATGAG | GGGAGAAGACGAATGTTTGC |
| RM537 | 4 | CCGTCCCTCTCTCTCCTTTC | ACAGGGAAACCATCCTCCTC |
| RM551 | 4 | AGCCCAGACTAGCATGATTG | GAAGGCGAGAAGGATCACAG |
| RM567 | 4 | ATCAGGGAAATCCTGAAGGG | GGAAGGAGCAATCACCACTG |
| RM1155 | 4 | AGGGAGTGTGGCAACTATGC | GGGAGGAGTGAGAAGGGATC |
| RM1236 | 4 | TCTCTCCGAAGGAGTGCTAGGG | CCAGCCTTAACACCATGTCTACG |
| RM2439 | 4 | ATGTTTAGATTCTTAGCACT | GCTCATATCCATATAAATGT |
| RM2441 | 4 | CCATGTGAGTTTAAATTCAC | ATTAACAGATGATGCAAATC |
| RM3263 | 4 | CCCCCTCCTTTAATTTGCAC | CTCCTGATCCTCATGGATGG |
| RM3473 | 4 | ATATTGGAAGGAGCAATCAC | CGTAATGTTGGTGAAGCAG |
| RM3839 | 4 | AATGGGACCAGAAAGCACAC | AAAAAGAGCATGGGGGCTAC |
| RM5030 | 4 | AGATTTTAGTGGTCCAAACA | ACTCAATTTCAACAATGGTG |
| RM5478 | 4 | ATCCAATGCGATGCTACTCC | CATCACGAGACCACGACAAG |
| RM5506 | 4 | AGGCGATGTTTGATCTCGAC | CTGGACGTACACACACGTACG |
| RM5635 | 4 | TCGCGTCGTAGCTGAACACTGC | TAGCTTGCTCTCCCTCTGCTTGC |
| RM5742 | 4 | GATCCTCAAACGGCCTCTGC | CCTTCAAAGTTTACTCACGCTCTGC |
| RM5757 | 4 | CTCTTCATCTTGAGCAGCAGCTTCG | CCGCCAACCTCCGATACTTCG |
| RM7181 | 4 | GTGAGAGTTCGTAGAAAGTGCC | GGATAAGCAAGATCCGTGAC |
| RM16994 | 4 | TGGCAGTACACACTACAGTACATGC | AGAGGGAGGAGAGAAAGGAAGG |
| RM13 | 5 | TCCAACATGGCAAGAGAGAG | GGTGGCATTCGATTCCAG |
| RM26 | 5 | GAGTCGACGAGCGGCAGA | CTGCGAGCGACGGTAACA |
| RM39 | 5 | GCCTCTCTCGTCTCCTTCCT | AATTCAAACTGCGGTGGC |
| RM153 | 5 | GCCTCGAGCATCATCATCAG | ATCAACCTGCACTTGCCTGG |
| RM164 | 5 | TCTTGCCCGTCACTGCAGATATCC | GCAGCCCTAATGCTACAATTCTTC |
| RM194 | 5 | GCCCTGCTTCTTGCCCACCACC | TCCAGGGAGGGCAAGGCTGAGC |
| RM274 | 5 | CCTCGCTTATGAGAGCTTCG | CTTCTCCATCACTCCCATGG |
| RM289 | 5 | TTCCATGGCACACAAGCC | CTGTGCACGAACTTCCAAAG |
| RM413 | 5 | GGCGATTCTTGGATGAAGAG | TCCCCACCAATCTTGTCTTC |
| RM421 | 5 | AGCTCAGGTGAAACATCCAC | ATCCAGAATCCATTGACCCC |
| RM440 | 5 | CATGCAACAACGTCACCTTC | ATGGTTGGTAGGCACCAAAG |
| RM574 | 5 | GGCGAATTCTTTGCACTTGG | ACGGTTTGGTAGGGTGTCAC |
| RM592 | 5 | TCTTTGGTATGAGGAACACC | AGAGATCCGGTTTGTTGTAA |
| RM598 | 5 | GAATCGCACACGTGATGAAC | ATGCGACTGATCGGTACTCC |
| RM3486 | 5 | TCTCTTTTCCCTCCTTTCCC | GGCCTGCAAGAGGAGAAAAC |
| RM3575 | 5 | CCTGGAATGATGATGGAAGG | GTTTTGCTTCCTGGAAGTGC |
| RM5642 | 5 | CCGTTTGTATGTAAGTACAG | AGAGAGAGAACTATTCGATG |
| RM6822 | 5 | AACTTGGGGCATTAACCTCC | AGGGGAGAGGAGAGTGAAGG |
| RM6952 | 5 | ACTCCATGACGGAATCGAAC | GGACATCAAAGGCACCATTC |
| RM7446 | 5 | TGAAGGCAGTTTCACTGACG | AGCCAAGAAGAAGAAAGGGG |
| RM30 | 6 | GGTTAGGCATCGTCACGG | TCACCTCACCACACGACACG |
| RM136 | 6 | GAGAGCTCAGCTGCTGCCTCTAGC | GAGGAGCGCCACGGTGTACGCC |
| RM170 | 6 | TCGCGCTTCTTCCTCGTCGACG | CCCGCTTGCAGAGGAAGCAGCC |
| RM204 | 6 | GTGACTGACTTGGTCATAGGG | GCTAGCCATGCTCTCGTACC |
| RM217 | 6 | ATCGCAGCAATGCCTCGT | GGGTGTGAACAAAGACAC |
| RM276 | 6 | CTCAACGTTGACACCTCGTG | TCCTCCATCGAGCAGTATCA |
| RM439 | 6 | TCATAACAGTCCACTCCCCC | TGGTACTCCATCATCCCATG |
| RM469 | 6 | AGCTGAACAAGCCCTGAAAG | GACTTGGGCAGTGTGACATG |
| RM528 | 6 | GGCATCCAATTTTACCCCTC | AAATGGAGCATGGAGGTCAC |
| RM539 | 6 | GAGCGTCCTTGTTAAAACCG | AGTAGGGTATCACGCATCCG |
| RM549 | 6 | ACGAACTGATCATATCCGCC | CTGTGGTTGATCCCTGAACC |
| RM584 | 6 | AGAAAGTGGATCAGGAAGGC | GATCCTGCAGGTAACCACAC |
| RM585 | 6 | CAGTCTTGCTCCGTTTGTTG | CTGTGACTGACTTGGTCATAGG |
| RM586 | 6 | ACCTCGCGTTATTAGGTACCC | GAGATACGCCAACGAGATACC |
| RM589 | 6 | ATCATGGTCGGTGGCTTAAC | CAGGTTCCAACCAGACACTG |
| RM1370 | 6 | AAACGAGAACCAACCGACAC | GGAGGGAGGAATGGGTACAC |
| RM3353 | 6 | AATGGTCGCCTCTCTCTCTG | GCTGGCATTGACCGTGTC |
| RM3827 | 6 | TAGTCCTCGAGGACGGATTG | CTGGCCTTTCTTCAATCTGC |
| RM4128 | 6 | AGTAACTCGATCAAACTAAC | AGAGTCCATATAGAATTTCA |
| RM5745 | 6 | ATGCCAAGTGGACGATGTAC | ACATGTGGGTAGTGGGATGG |
| RM6536 | 6 | GAGCTGCCGCTAAGGTTCC | AAAATCCACTCCCGCTTCC |
| RM7193 | 6 | ATGTGGGAATTTCTAGCCCC | CCCTAGTTTTCCAAATGGCC |
| RM8072 | 6 | GATCACTCAGGTCATCCATTC | AATCAGAGAGGCTAAAGACAATAAT |
| RM8120 | 6 | AAGATGAGTAAGTTTAATTGACCTGAT | GAAAGCCTATCACTATATATCTAACTAAGC |
| RM8200 | 6 | CTAGCCATGCTCTCGTACC | CAACTTACTGTGACTGACTTGG |
| RM125 | 7 | ATCAGCAGCCATGGCAGCGACC | AGGGGATCATGTGCCGAAGGCC |
| RM182 | 7 | TGGGATGCAGAGTGCAGTTGGC | CGCAGGCACGGTGCCTTGTAAG |
| RM336 | 7 | CTTACAGAGAAACGGCATCG | GCTGGTTTGTTTCAGGTTCG |
| RM346 | 7 | CGAGAGAGCCCATAACTACG | ACAAGACGACGAGGAGGGAC |
| RM420 | 7 | GGACAGAATGTGAAGACAGTCG | ACTAATCCACCAACGCATCC |
| RM429 | 7 | TCCCTCCAGCAATGTCTTTC | CCTTCATCTTGCTTTCCACC |
| RM481 | 7 | TAGCTAGCCGATTGAATGGC | CTCCACCTCCTATGTTGTTG |
| RM505 | 7 | AGAGTTATGAGCCGGGTGTG | GATTTGGCGATCTTAGCAGC |
| RM542 | 7 | TGAATCAAGCCCCTCACTAC | CTGCAACGAGTAAGGCAGAG |
| RM560 | 7 | GCAGGAGGAACAGAATCAGC | AGCCCGTGATACGGTGATAG |
| RM1134 | 7 | ACACCCAACTTTTCTCACGC | AGCTAGGGTTTCGATCTCCC |
| RM1243 | 7 | TTTCGGGAGGGATTATGACC | GTGACCCCCGATACAAACAC |
| RM2256 | 7 | GTGCTTGCATATAACCTATA | AGATCAACCTTCTTATTCAG |
| RM3394 | 7 | CCCTTACGTGCAGTACATTG | ATGCAGGCTACTTACTAGCG |
| RM3753 | 7 | CCGAGATTAAAACTCAAATG | ACTTTTGTAGATTCCTCGAA |
| RM5426 | 7 | GAGAGTAGTGGCTGTTCCGC | GCGGTTTCGATCTCTACGAG |
| RM5508 | 7 | TCGCACACTAGCTCGATCAG | TGGTCCTCTTCTCCATCCAG |
| RM6394 | 7 | GGGCACACTGCTTGATCC | TCGACGTCCACGATGACTAC |
| RM7601 | 7 | GCCTCGCTGTCGCTAATATC | CAGCCTCTCCTTGTGTTGTG |
| RM8008 | 7 | ACCAAATCTTTAATTCAATG | CCTTCTTCCTTCCTAGTCTA |
| RM72 | 8 | CCGGCGATAAAACAATGAG | GCATCGGTCCTAACTAAGGG |
| RM152 | 8 | GAAACCACCACACCTCACCG | CCGTAGACCTTCTTGAAGTAG |
| RM210 | 8 | TCACATTCGGTGGCATTG | CGAGGATGGTTGTTCACTTG |
| RM331 | 8 | GAACCAGAGGACAAAAATGC | CATCATACATTTGCAGCCAG |
| RM337 | 8 | GTAGGAAAGGAAGGGCAGAG | CGATAGATAGCTAGATGTGGCC |
| RM339 | 8 | GTAATCGATGCTGTGGGAAG | GAGTCATGTGATAGCCGATATG |
| RM407 | 8 | GATTGAGGAGACGAGCCATC | CTTTTTCAGATCTGCGCTCC |
| RM506 | 8 | CGAGCTAACTTCCGTTCTGG | GCTACTTGGGTAGCTGACCG |
| RM515 | 8 | TAGGACGACCAAAGGGTGAG | TGGCCTGCTCTCTCTCTCTC |
| RM1109 | 8 | ATGATCGATCGATCCAGGAG | GGACCAGTTCACTGTGCATG |
| RM1376 | 8 | CATGTGTGATGACTGACAGG | GGTGCTGTGATGATTCTTTC |
| RM3181 | 8 | TTAGCGAAGCAAACCCTCAC | GAGAAGGCTTTGCTTGCCTC |
| RM3395 | 8 | ACCTCATGTCCAGGTGGAAG | AGATTAGTGCCATGGCAAGG |
| RM3572 | 8 | AGTGCTGTCTGGTTTTTGGC | CCCCTCCCTTTCTTTCTTTG |
| RM4955 | 8 | GCATCCAGCAATATAATCAA | CAAGGATTTTGTTAAGTGGG |
| RM5637 | 8 | CAACTCCAACGACGATGAAC | TGGTGAAGTGGAGTGGAGTG |
| RM6925 | 8 | TGAGAGGACGCTTGAAGAGG | GCACCTAGTGACTGAAGGTTG |
| RM6999 | 8 | TTATCTGGGATCCATCGAGC | GTGAATTTCCTTGGAGGGAC |
| RM7027 | 8 | AGGACCTGGACTTTATGGGC | CCTGCACTGCTCCACAGTAC |
| RM8040 | 8 | GTGTATTGTGTAAATATGAC | AGATTATGAATTACTAGGAC |
| RM8271 | 8 | TCTTGAGAAATCTGCCATTC | ACTGATGTGCATTTCGTC |
| RM22242 | 8 | TGTTCCACATGTTGTACTCCATCC | ACGTTCAGTACAGTCGCCAACG |
| RM23386 | 8 | AGGTTGACCTGTGTGAGTAGCAAGG | ACATCGCCAACCATCTCAAGG |
| RM23419 | 8 | ACCTGAGCTCGATGGTTCTTTCC | GCTCCCACCAAGTATTCCTATCG |
| RM23442 | 8 | TGATACAAGCCATCCCTCACC | GAGAGGAGGGATTTGCACTGG |
| RM41 | 9 | AAGTCTAGTTTGCCTCCC | AATTTCTACGTCGTCGGGC |
| RM107 | 9 | AGATCGAAGCATCGCGCCCGAG | ACTGCGTCCTCTGGGTTCCCGG |
| RM108 | 9 | TCTCTTGCGCGCACACTGGCAC | CGTGCACCACCACCACCACCAC |
| RM219 | 9 | CGTCGGATGATGTAAAGCCT | CATATCGGCATTCGCCTG |
| RM245 | 9 | ATGCCGCCAGTGAATAGC | CTGAGAATCCAATTATCTGGGG |
| RM434 | 9 | GCCTCATCCCTCTAACCCTC | CAAGAAAGATCAGTGCGTGG |
| RM444 | 9 | GCTCCACCTGCTTAAGCATC | TGAAGACCATGTTCTGCAGG |
| RM460 | 9 | TGATCGACAGCGTTCTTGAC | GCCTGGCCCACATAATTAAG |
| RM553 | 9 | AACTCCACATGATTCCACCC | GAGAAGGTGGTTGCAGAAGC |
| RM1099 | 9 | CTCGGCGAATCAGAGAAGAC | ATCCTAACGTGCCTATCCCC |
| RM1553 | 9 | AATTAGAGGGTCCACATGTC | ATTACCCTCATTTTCTACGC |
| RM3912 | 9 | TGTGTGTGCCCGATCTAC | CCTCTCGATGAGCATTCC |
| RM5519 | 9 | GGCCTTTGGTTACCCCTAAC | TGTACAGCAAAAGCAACCCC |
| RM5535 | 9 | CGTTCGTGGAGTGGTATGTG | CATACCGAAGTGAGGAACTCG |
| RM7424 | 9 | AGAAGCCCATCTAGCAGCAG | TCAAGCTAGCCACACAGCTG |
| RM171 | 10 | AACGCGAGGACACGTACTTAC | ACGAGATACGTACGCCTTTG |
| RM187 | 10 | CCAAGGGAAAGATGCGACAATTG | GTGGACGCTTTATATTATGGG |
| RM216 | 10 | GCATGGCCGATGGTAAAG | TGTATAAAACCACACGGCCA |
| RM244 | 10 | CCGACTGTTCGTCCTTATCA | CTGCTCTCGGGTGAACGT |
| RM311 | 10 | TGGTAGTATAGGTACTAAACAT | TCCTATACACATACAAACATAC |
| RM333 | 10 | GTACGACTACGAGTGTCACCAA | GTCTTCGCGATCACTCGC |
| RM467 | 10 | GGTCTCTCTCTCTCTCTCTCTCTC | CTCCTGACAATTCAACTGCG |
| RM591 | 10 | CTAGCTAGCTGGCACCAGTG | TGGAGTCCGTGTTGTAGTCG |
| RM596 | 10 | ATCTACACGGACGAATTGCC | AGAAGCTTCAGCCTCTGCAG |
| RM2824 | 10 | CATTCCATAATCTATCCACG | ACTCAATTTCAACAATGGTG |
| RM3283 | 10 | CCCGTTAAAAGGGAAACTCC | CGAACTCCTAGACTCCACCG |
| RM3451 | 10 | CGGCGAGATAACAATTCTCC | GCGTGATGATATGGTATCGG |
| RM3590 | 10 | GTCTTGCTGCACCCTCTTTC | CACCACTGCACACAATCCAC |
| RM3717 | 10 | AGCTCTACCTTTGCTGTCGG | AACTCCCTAGACCCACCTGC |
| RM4455 | 10 | CTCTCAAAGAACTAGGACTC | GAGAAGGTATGATAACCAAT |
| RM5348 | 10 | AATCCGATAGGAGTACCGCC | AAGTGTATGGGCTGGAATGG |
| RM5373 | 10 | AAACGGTGGTCTCATCATTCTGC | TCCCATCAGTCGTCAGATATTGC |
| RM6132 | 10 | ACTAAACCCAGCAAAGCCAAACG | GGCTTAGGCCCACAGTTCTTGC |
| RM6469 | 10 | CAGGAGTCGTCGTCGATGTGG | AAGGAGGGTTCTTTGGTGTACCG |
| RM6868 | 10 | TGAACATGCCGAGGAAGC | ATATAGAACCCAAAGCCCCC |
| RM21 | 11 | ACAGTATTCCGTAGGCACGG | GCTCCATGAGGGTGGTAGAG |
| RM116 | 11 | TCACGCACAGCGTGCCGTTCTC | CAAGATCAAGCCATGAAAGGAGGG |
| RM144 | 11 | TGCCCTGGCGCAAATTTGATCC | GCTAGAGGAGATCAGATGGTAGTGCATG |
| RM167 | 11 | GATCCAGCGTGAGGAACACGT | AGTCCGACCACAAGGTGCGTTGTC |
| RM187 | 11 | CCAAGGGAAAGATGCGACAATTG | GTGGACGCTTTATATTATGGG |
| RM209 | 11 | ATATGAGTTGCTGTCGTGCG | CAACTTGCATCCTCCCCTCC |
| RM224 | 11 | ATCGATCGATCTTCACGAGG | TGCTATAAAAGGCATTCGGG |
| RM229 | 11 | CACTCACACGAACGACTGAC | CGCAGGTTCTTGTGAAATGT |
| RM254 | 11 | AGCCCCGAATAAATCCACCT | CTGGAGGAGCATTTGGTAGC |
| RM287 | 11 | TTCCCTGTTAAGAGAGAAATC | GTGTATTTGGTGAAAGCAAC |
| RM332 | 11 | GCGAAGGCGAAGGTGAAG | CATGAGTGATCTCACTCACCC |
| RM441 | 11 | ACACCAGAGAGAGAGAGAGAGAG | TCTGCAACGGCTGATAGATG |
| RM457 | 11 | CTCCAGCATGGCCTTTCTAC | ACCTGATGGTCAAAGATGGG |
| RM1124 | 11 | AAGCTATCCCCCTTTTTGGC | AGGGATCGGTAGACCCAATC |
| RM1812 | 11 | CAGCTAGTGAGCTCCTAGTG | GCTAACCCACCAACTTATTC |
| RM3133 | 11 | TCAATAGACACACGGGCATG | CGATTTTGCTCACTGCACAG |
| RM3717 | 11 | AGCTCTACCTTTGCTGTCGG | AACTCCCTAGACCCACCTGC |
| RM5997 | 11 | GCGACGACGAAGAAGCTAAC | CCCATCGATAGGGTTTCCTC |
| RM6085 | 11 | GGTGAGAGATGGCTAAAGCG | CATCGCCTCTAGCACCTCC |
| RM6091 | 11 | GCTGTCCTGTCCTTGAATCC | TGGTAGGCTGGTGACATGC |
| RM7315 | 11 | CACAAAGGCGTGTGGGTTAG | GAGTCACGGGATGTTGCC |
| RM7443 | 11 | TGCTGCGTGTTACTTTGGTG | AACCCTTCATCAGGCTACGC |
| RM17 | 12 | TGCCCTGTTATTTTCTTCTCTC | GGTGATCCTTTCCCATTTCA |
| RM83 | 12 | ACTCGATGACAAGTTGAGG | CACCTAGACACGATCGAG |
| RM179 | 12 | CCCCATTAGTCCACTCCACCACC | CCAATCAGCCTCATGCCTCCCC |
| RM260 | 12 | ACTCCACTATGACCCAGAG | GAACAATCCCTTCTACGATCG |
| RM448 | 12 | TCTGATCTTGATGCAGGCAC | TCTCCCGATTTGGACAGATC |
| RM453 | 12 | CGCATCTCTCTCCCTTATCG | CTCTCCTCCTCGTTGTCGTC |
| RM511 | 12 | CTTCGATCCGGTGACGAC | AACGAAAGCGAAGCTGTCTC |
| RM519 | 12 | AGAGAGCCCCTAAATTTCCG | AGGTACGCTCACCTGTGGAC |
| RM1246 | 12 | AGCTCGATCCCCTAGCTCTC | TTGGAGAAGGTCACCTGCC |
| RM1880 | 12 | ACCACTAAATAAGCACATAC | GGCATCATACATTAAAATAC |
| RM3739 | 12 | AGTTGCGCAGCTAATCGATC | AAGATCCAACGGGTTCTGTG |
| RM5195 | 12 | TCTCTGTTCTTGGGTTTAAC | CCGACCAATTTTATTAAGAT |
| RM5479 | 12 | CTAAGCTCACCATAGCAATC | ATACACTTCTCCCCTCTCTG |
| RM5609 | 12 | CGCCAGTGTCGAATATGATG | TCTTGGTGCAGTAGGTGCAC |
| RM5851 | 12 | GCTGTCGGGGATGTAATACG | GCTTTGCGGCTGGTTAATTG |
| RM7018 | 12 | CATCGTTGACCGCTGCTC | AATAAACAGCACGTGCTCCC |
| RM7195 | 12 | GCCACTGGAAACAATTGAAACG | CGCTTTGTCCTTGTGTAACTACCG |
| RM28438 | 12 | GTTCGTGAGCCACAACAAATCC | GTTAAATGCTCCACCAAACACACC |

**Table S9** **Genetic background screening information of the design breeding lines.**

| Chromosome number | No. of  markers | Average interval between markers(kb) | | No. of polymorphic markers | Percentage of chromosome segments from recurrent parents  MD1 MD2 MD3 | | |
| --- | --- | --- | --- | --- | --- | --- | --- |
| 1 | 75 | 576.9 | 25 | | 88.9 | 82.4 | 78.3 |
| 2 | 61 | 589.1 | 20 | | 85.3 | 86.5 | 87.2 |
| 3 | 62 | 587.3 | 18 | | 85.7 | 92.5 | 85.7 |
| 4 | 62 | 572.6 | 30 | | 93.3 | 76.9 | 78.1 |
| 5 | 56 | 534.9 | 20 | | 88.3 | 87.5 | 88.3 |
| 6 | 58 | 538.7 | 25 | | 82.7 | 84.6 | 83.5 |
| 7 | 56 | 530.3 | 20 | | 90.5 | 85.2 | 85.2 |
| 8 |  | 526.7 | 25 | | 80.3 | 70.5 | 76.1 |
| 9 | 42 | 547.9 | 15 | | 83.3 | 90.7 | 80.3 |
| 10 | 44 | 527.4 | 20 | | 75.1 | 75.1 | 80.8 |
| 11 | 52 | 558.1 | 22 | | 88.6 | 80.0 | 80.0 |
| 12 | 50 | 550.6 | 18 | | 78.2 | 78.2 | 83.8 |
| Total(average) | 672 | 553.4 | 258 | | 85.0 | 82.5 | 82.4 |

**Reference**

Guo J., Xu C., Wu D., Zhao Y., Qiu Y., Wang X., Ouyang Y., Cai B., Liu X., Jing S., Shangguan X., Wang H., Ma Y., Hu L., Wu Y., Shi S., Wang W., Zhu L., Xu X., Chen R., Feng Y., Du B. & He G. (2018) Bph6 encodes an exocyst-localized protein and confers broad resistance to planthoppers in rice. *Nat Genet* **50**, 297-306.

Tian Z., Qian Q., Liu Q., Yan M., Liu X., Yan C., Liu G., Gao Z., Tang S., Zeng D., Wang Y., Yu J., Gu M. & Li J. (2009) Allelic diversities in rice starch biosynthesis lead to a diverse array of rice eating and cooking qualities. *Proc Natl Acad Sci U S A* **106**, 21760-21765.

Wang S., Li S., Liu Q., Wu K., Zhang J., Wang Y., Chen X., Zhang Y., Gao C., Wang F., Huang H. & Fu X. (2015) The OsSPL16-GW7 regulatory module determines grain shape and simultaneously improves rice yield and grain quality. *Nat Genet* **47**, 949-954.

Zeng D., Tian Z., Rao Y., Dong G., Yang Y., Huang L., Leng Y., Xu J., Sun C., Zhang G., Hu J., Zhu L., Gao Z., Hu X., Guo L., Xiong G., Wang Y., Li J. & Qian Q. (2017) Rational design of high-yield and superior-quality rice. *Nat Plants* **3**, 17031.

Zhao Y., Huang J., Wang Z., Jing S., Wang Y., Ouyang Y., Cai B., Xin X.F., Liu X., Zhang C., Pan Y., Ma R., Li Q., Jiang W., Zeng Y., Shangguan X., Wang H., Du B., Zhu L., Xu X., Feng Y.Q., He S.Y., Chen R., Zhang Q. & He G. (2016) Allelic diversity in an NLR gene BPH9 enables rice to combat planthopper variation. *Proc Natl Acad Sci U S A* **113**, 12850-12858.
